# Uterotonics for prevention of postpartum haemorrhage: EN-BIRTH multi-country validation study

**DOI:** 10.1186/s12884-020-03420-x

**Published:** 2021-03-26

**Authors:** Harriet Ruysen, Josephine Shabani, Claudia Hanson, Louise T. Day, Andrea B. Pembe, Kimberly Peven, Qazi Sadeq-ur Rahman, Nishant Thakur, Kizito Shirima, Tazeen Tahsina, Rejina Gurung, Menna Narcis Tarimo, Allisyn C. Moran, Joy E. Lawn, EN-BIRTH Study Group, Ahmed Ehsanur Rahman, Sojib Bin Zaman, Shafiqul Ameen, Tanvir Hossain, Abu Bakkar Siddique, Aniqa Tasnim Hossain, Tapas Mazumder, Jasmin Khan, Taqbir Us Samad Talha, Rajib Haider, Md. Hafizur Rahman, Anisuddin Ahmed, Shams El Arifeen, Omkar Basnet, Avinash K. Sunny, Anjani Kumar Jha, Bijay Jha, Ram Chandra Bastola, Rajendra Paudel, Asmita Paudel, K. C. Ashish, Nahya Salim, Donat Shamba, Kizito Shirima, Godfrey Mbaruku, Honorati Masanja, Harriet Ruysen, Vladimir Sergeevich Gordeev, Georgia R. Gore-Langton, Dorothy Boggs, Stefanie Kong, Angela Baschieri, Simon Cousens

**Affiliations:** 1 Centre for Maternal, Adolescent, Reproductive & Child Health (MARCH), London School of Hygiene & Tropical Medicine (LSHTM), London, UK; 2 Department of Health Systems, Impact Evaluation and Policy, Ifakara Health Institute (IHI), Dar Es Salaam, Tanzania; 3 Public Health Sciences - Global Health - Health Systems and Policy, Karolinska Institutet, Stockholm, Sweden; 4 Department of Obstetrics and Gynaecology, Muhimbili University of Health and Allied Sciences (MUHAS), Dar Es Salaam, Tanzania; 5 Florence Nightingale Faculty of Nursing, Midwifery & Palliative Care, King’s College London, London, UK; 6 Maternal and Child Health Division, International Centre for Diarrhoeal Disease Research, Bangladesh (icddr,b), Dhaka, Bangladesh; 7 Research division, Golden Community, Lalitpur, Nepal; 8 Department of Maternal, Newborn, Child and Adolescent Health, World Health Organization, Geneva, Switzerland

**Keywords:** Birth, Maternal, Coverage, Validity, Survey, Hospital records, Health management systems, Uterotonics, Postpartum haemorrhage

## Abstract

**Background:**

Postpartum haemorrhage (PPH) is a leading cause of preventable maternal mortality worldwide. The World Health Organization (WHO) recommends uterotonic administration for every woman after birth to prevent PPH. There are no standardised data collected in large-scale measurement platforms. The *Every Newborn* Birth Indicators Research Tracking in Hospitals (EN-BIRTH) is an observational study to assess the validity of measurement of maternal and newborn indicators, and this paper reports findings regarding measurement of coverage and quality for uterotonics.

**Methods:**

The EN-BIRTH study took place in five hospitals in Bangladesh, Nepal and Tanzania, from July 2017 to July 2018. Clinical observers collected tablet-based, time-stamped data. We compared observation data for uterotonics to routine hospital register-records and women’s report at exit-interview survey. We analysed the coverage and quality gap for timing and dose of administration. The register design was evaluated against gap analyses and qualitative interview data assessing the barriers and enablers to data recording and use.

**Results:**

Observed uterotonic coverage was high in all five hospitals (> 99%, 95% CI 98.7–99.8%). Survey-report underestimated coverage (79.5 to 91.7%). “Don’t know” replies varied (2.1 to 14.4%) and were higher after caesarean (3.7 to 59.3%). Overall, there was low accuracy in survey data for details of uterotonic administration (type and timing). Register-recorded coverage varied in four hospitals capturing uterotonics in a specific column (21.6, 64.5, 97.6, 99.4%). The average coverage measurement gap was 18.1% for register-recorded and 6.0% for survey-reported coverage. Uterotonics were given to 15.9% of women within the “right time” (1 min) and 69.8% within 3 min. Women’s report of knowing the purpose of uterotonics after birth ranged from 0.4 to 64.9% between hospitals. Enabling register design and adequate staffing were reported to improve routine recording.

**Conclusions:**

Routine registers have potential to track uterotonic coverage – register data were highly accurate in two EN-BIRTH hospitals, compared to consistently underestimated coverage by survey-report. Although uterotonic coverage was high, there were gaps in observed quality for timing and dose. Standardisation of register design and implementation could improve data quality and data flow from registers into health management information reporting systems, and requires further assessment.

**Supplementary Information:**

**Supplementary information** accompanies this paper at 10.1186/s12884-020-03420-x.

## Key findings

**Table:** 

**What is known and what is new about this study?** • Administration of prophylactic uterotonics immediately after birth is an evidence-based intervention with the potential to reduce postpartum haemorrhage (PPH) related deaths by half, yet there are no reliable data tracking current coverage at national or global level for most low and middle-income countries (LMICs). • EN-BIRTH is the first and largest observational study (*n* = 23,015 women) with mixed methods to assess validity of uterotonic measurement around the time of birth in three LMICs. Custom-built tablet-based software generated time-stamped observation data. Qualitative research explored barriers and enablers to inform improvements for routine register recording of uterotonic use. **Survey-what did we find and what does it mean?** • Our findings show women’s reports about care received around the time of birth underestimate uterotonics coverage; this aligns with results from previous studies. • There was low accuracy in survey data for details of uterotonic administration (type of drug and timing of administration). We do not recommend the addition of a uterotonic indicator to household survey platforms. • “Don’t know” responses were highest for women having a caesarean birth. **Register-what did we find and what does it mean?** • Register design was critical: one did not capture uterotonics at all. • When uterotonics were recorded in specific columns, coverage was accurately measured in two hospitals but underestimated in two hospitals, suggesting that good register design is necessary, but not sufficient to achieve high quality data. **Gap analysis for quality of care and measurement** • Uterotonic coverage was high (> 99%) in these five hospitals. • Actionable gaps were identified for timing—only 15.9% of women received uterotonics within the recommended 1 min, and 69.8% of women within 3 min. • The correct dose of oxytocin was received by 63.3% of women. **What next and research gaps?** • Uterotonic coverage was high, so we need to move beyond coverage, and measure the quality of uterotonic administration. Data sources such as local audits—as well as service readiness or health facility assessments monitoring drug quality, stock management and provider practices—are needed. • Further research to explore data flow and quality at different levels of the HMIS, and measures of effective coverage, is also warranted. • Registers have potential to accurately capture provision of uterotonics and could provide regular data with standardised design and implementation.	

## Background

An estimated 295 000 maternal deaths occur annually worldwide, 99% are in low and middle income countries (LMICs) [1]. Approximately one-quarter of maternal deaths are caused by haemorrhage, with postpartum haemorrhage (PPH) estimated to affect around 7 million women each year [2, 3]. Administration of prophylactic uterotonics immediately after birth is an evidence-based intervention with potential to halve PPH-related deaths [4]. The World Health Organization (WHO) recommends provision of prophylactic uterotonics for every woman during the third stage of labour [5]. Five drugs are available for PPH prevention: oxytocin, carbetocin, ergometrine, misoprostol, and prostaglandin. An intramuscular (IM) injection of oxytocin plus ergometrine is most effective, although oxytocin alone is currently the most widely used uterotonic for facility births [4]. Despite uterotonics being prioritised by WHO as an essential intervention, there are currently no national or global level data to track coverage. Several estimates based on expert opinion suggest low coverage [6, 7], and one study found coverage under 50% in three settings with low facility-birth rates [8].

Data on coverage, equity and quality of care are fundamental to achieving Universal Health Coverage and driving progress towards meeting the Sustainable Development Goals for maternal and neonatal mortality, as well as addressing morbidities, by 2030 [9, 10]. Quality of care at birth is prioritised by both *Every Newborn* and Ending Preventable Maternal Mortality (EPMM) strategies [11–13]. The *Every Newborn* Action Plan, passed by all United Nations member states and agreed by more than 80 development partners, includes an ambitious measurement improvement roadmap with an urgent focus on validating indicators for selected maternal and newborn care interventions [13, 14].

Coverage is defined as the proportion of individuals receiving an intervention (numerator: ‘*number of women receiving prophylactic uterotonics immediately after birth in a health facility*) from among the population in need of that intervention (denominator: *all women giving birth in the facility’*) [15, 16]. The use of live births as the denominator is common for many maternal health indicators such as place of birth, skilled attendance or caesarean section [6], but should be carefully evaluated for appropriateness against each indicator.

Population-based surveys such as the Demographic and Health Survey (DHS) and Multiple Indicator Cluster Survey (MICS) remain the major data sources for pregnancy outcomes and coverage of care data for the 75% of births occurring in LMICs [17–19]. Currently, there is no uterotonic indicator measured in core survey modules for DHS or MICS. Previous research to assess validity of surveys suggest women do not accurately report uterotonic administration [20–23]. In two of five studies, agreed cut-offs for population-level validity were met, but none met individual-level validity thresholds [20, 21] (Additional file 1). This is compatible with further evidence suggesting that asking women about clinical interventions provided during or immediately after birth is not reliable [20–24].

Facility-based births in LMICs have increased dramatically in the last decade, now reaching four out of every five births [25]. Data recorded in facility registers and aggregated as part of health management information systems (HMIS) offer an alternative measurement platform, which could provide more frequent information if concerns about data quality and completeness are addressed [26]. Only one previous observational study (*n* = 1867) in Nigeria has assessed register-recorded accuracy compared with observer-assessed coverage for uterotonics [27]. They found accurate measurement with nearly complete agreement between register-recorded and observer-assessed data for uterotonics, but were unable to analyse individual-level validity due to high intervention prevalence [27]. In a descriptive assessment of birth registers in 37 countries, only 16 were tracking uterotonics use in any routine record, including maternity registers, birth records, or electronic data platforms [7].

The *Every Newborn* – Birth Indicators Research Tracking in Hospitals (EN-BIRTH) study was an observational study of > 23,000 hospital births in three countries (Tanzania, Bangladesh and Nepal). The detailed protocol as well as overall validity results, are reported elsewhere [15, 28].

## Objectives

This paper is part of a supplement based on the EN-BIRTH multi-country validation study, *‘Informing measurement of coverage and quality of maternal and newborn care’*, and focuses on uterotonic provision with four objectives:

1. **Assess NUMERATOR accuracy/validity** of uterotonic coverage measurement using exit survey of women’s report, and routine labour ward registers compared to direct observation (gold standard).
2. **Compare DENOMINATOR options for uterotonic coverage:** including live births, or total births (live births and stillbirths).
3. **Analyse GAPS in coverage and quality of care, and measurement for uterotonics:** coverage and quality gaps relating to provision of care (right time, right drug, and right dose) and experience of care (survey report of reason for uterotonics given).
4. **Evaluate BARRIERS AND ENABLERS** to routine labour ward register recording for uterotonics through qualitative interviews regarding register design filling and use.

## Methods

**Fig. 1:**
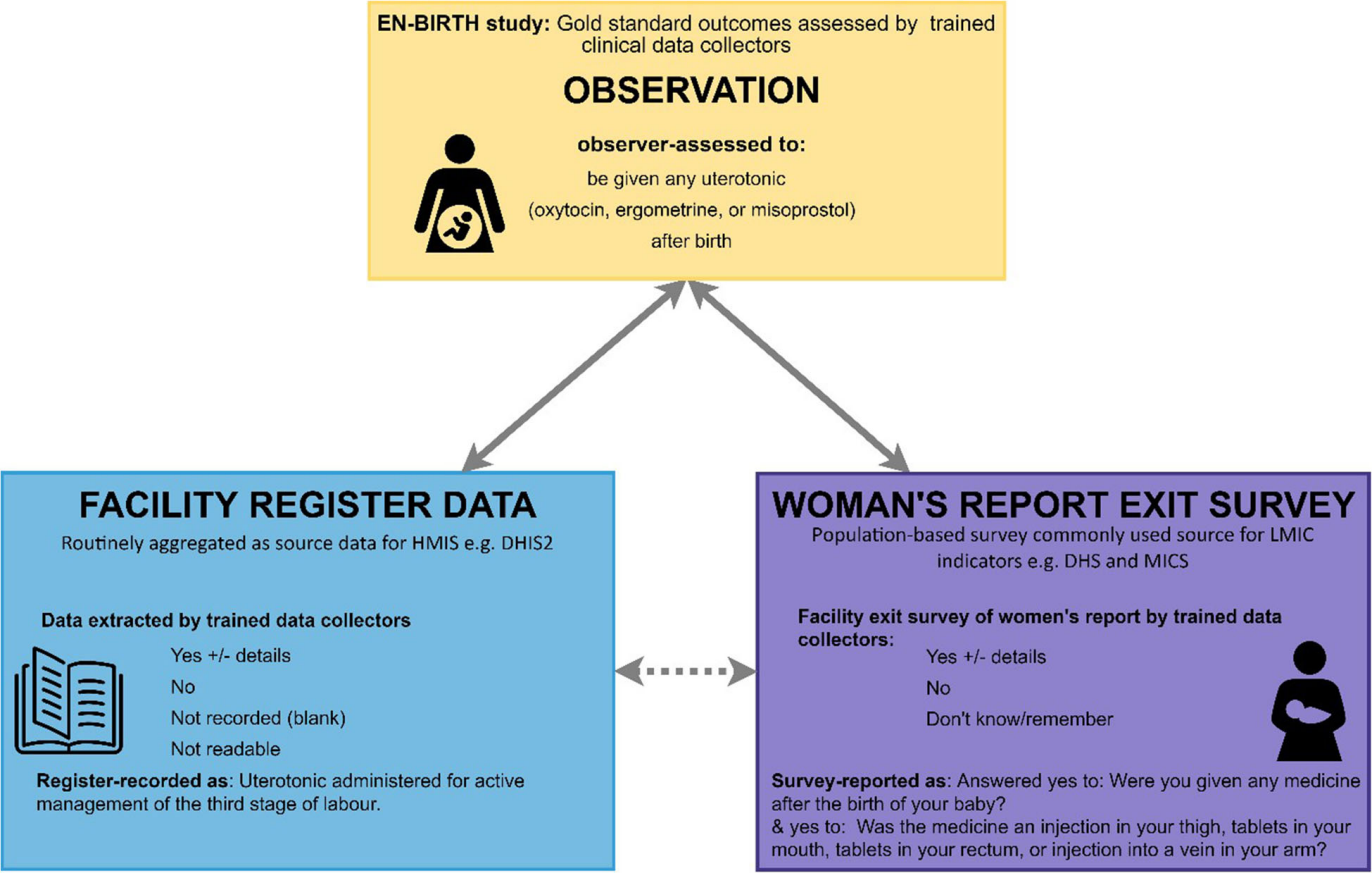
Uterotonics: validation design, EN-BIRTH study. EN-BIRTH validation Design comparing observation gold standard with register-recorded and women’s report on exit survey. EN-BIRTH data collection tools (observation checklist, register data extraction tool and exit-interview survey tool) are published separately [15, 29]

EN-BIRTH study compared observation of uterotonic administration for prevention of PPH (gold standard) to coverage measured by women’s report at exit-interview survey, and routine register records (Fig. 1). Gold standard data were collected by trained clinical researchers covering 24 h per day and using a custom-built android tablet-based software application [15].

Five comprehensive emergency obstetric care (CEmOC) hospitals in three study countries were included because they were implementing the selected interventions: Maternal and Child Health Training Institute, Azimpur and Kushtia General Hospital in Bangladesh (BD), Pokhara Academy Health Sciences in Nepal (NP), and Muhimbili National Hospital and Temeke District Hospital in Tanzania (TZ). Participants were consenting women admitted to the labour and birth wards in the five study sites. Data collection was undertaken between July 2017 and July 2018. This study was granted ethical approval by institutional review boards in all operating countries in addition to the London School of Hygiene & Tropical Medicine (Additional file 2). Results are reported in accordance with STROBE statement checklists for cross-sectional studies (Additional file 3).

Labour ward registers varied in design between the five sites. Nepal had no uterotonics column. The original Bangladesh hospital registers, and an additional ‘midwifery book’ maintained in Muhimbili, had a non-specific column option (such as ‘drugs’). Bangladesh registers were updated to a standardised national register during the study (Additional file 4). Tanzanian and the updated Bangladesh registers used for this analysis had a specific column for third stage management, labelled ‘AMTSL’ (active management of the third stage). In Bangladesh, staff ticked the column if AMTSL (including uterotonic administration) was considered done, and left the column blank for not done. The AMTSL column in Tanzania was completed with an “O, E or M” denoting oxytocin, ergometrine or misoprostol administration. There was a further column in the Tanzania registers where staff could write “yes” if any type of uterotonic was administered, or “no” if no uterotonic was administered. Full details of register design and use are available in Additional file 5.

One year of pre-study register data were extracted and compared to one-year of during-after study register records to assess if the presence of external researchers in the hospital affected register recording practice [28, 30]. To determine reliability of the observational data, Cohen’s Kappa coefficients of agreement were calculated for a 5% subset of cases where study supervisors simultaneously observed/extracted data for comparison with data collector’s findings (Additional file 6) [28].

### Objective 1: Numerator validation

**Fig. 2:**
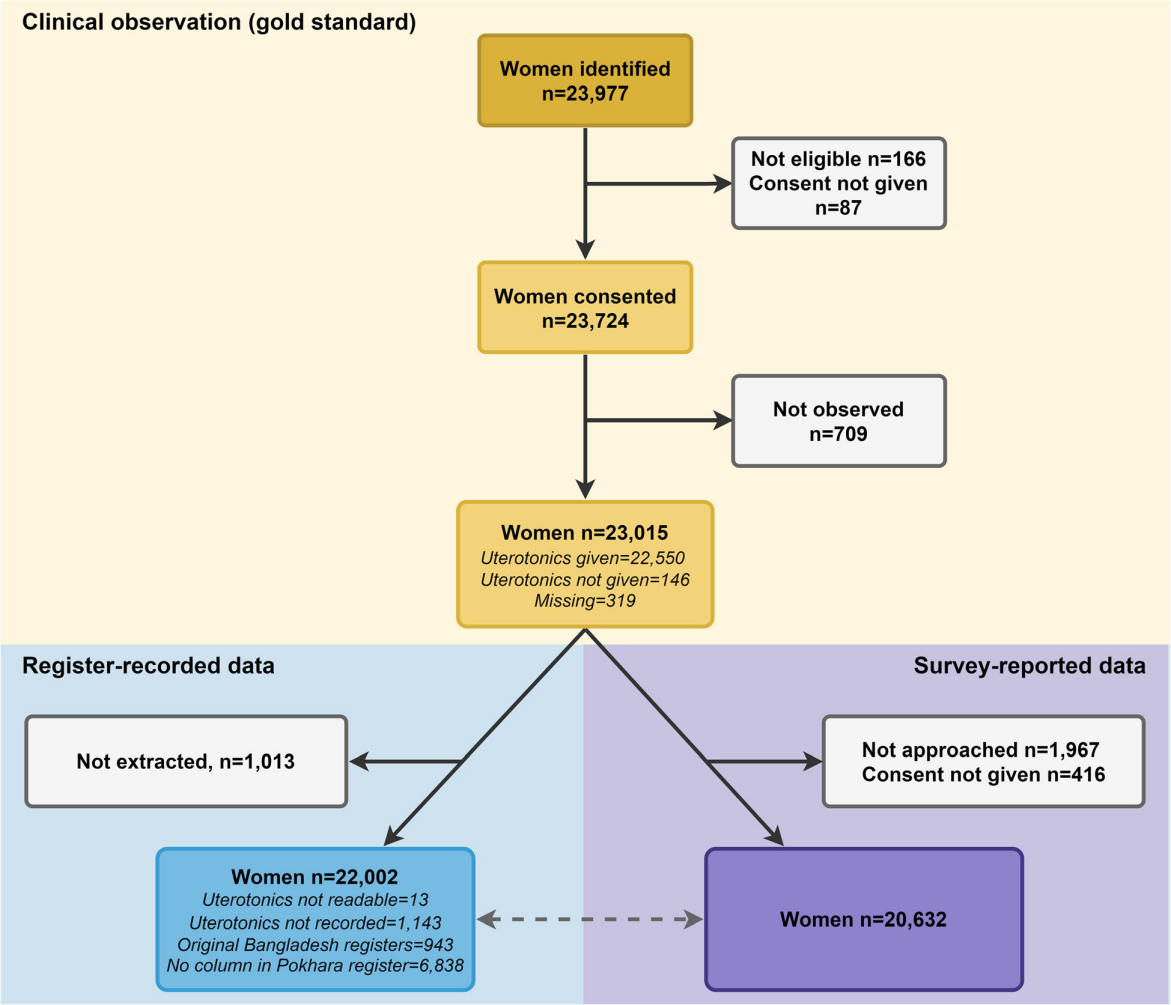
Flow diagram for uterotonic cases, EN-BIRTH study (*n* = 23,015)

We assessed the performance of a range of individual and combined exit-survey questions around uterotonic administration for prevention of PPH, compared to observer-assessed practice (Fig. 2). All results were stratified by mode of birth (vaginal births and caesareans) and presented by individual site, and overall. For indicators which had ≥10 counts in both columns of the 2 × 2 table, we calculated percent agreement, sensitivity, and specificity, positive and negative predictive values, area under the receiver operating curve, and inflation factor. We combined hospital data using random effects meta-analysis [28]. The percentage of women answering “don’t know” to survey questions was calculated and analysed in two ways: “don’t know” considered as “no” and with “don’t know” excluded [28]. If there were missing data elements for the numerator or denominator, the participant was excluded from the relevant sample. Nepal was excluded from register-recorded validation calculations given the absence of a uterotonic column. Exit-interview indicator combinations were explored using descriptive analysis comparing women’s report for different combined indicator options with observation data (Additional file 7). Quantitative analyses were undertaken using StataCorp: Stata Statistical Software (Release 16. In. College Station, TX; 2019).

### Objective 2: Denominator comparison

The denominator was all women who gave birth; however, we also calculated coverage using live birth and total birth denominator options for observer-assessed uterotonic coverage data. Descriptive analysis was used to compare these results.

### Objective 3: Gap analysis for coverage and quality of care and measurement

We analysed four gaps for uterotonic administration: 1) Coverage gap between the all-birth target population and observed uterotonic coverage. 2) Quality of care gap between *any* observed uterotonic coverage, and *high quality* uterotonic coverage (within the right time < 1 or < 3 min, at the right dose 10 international units (IU) oxytocin). 3) Measurement gap for register-records. 4) Measurement gap for survey reports. Results were stratified by site and by mode of birth, univariate logistic regression was used to explore the association between timing of uterotonic administration and mode of birth.

### Objective 4: Barriers and enablers to data collection

Qualitative data collection tools for focus group interviews were informed by the Performance of Routine Information System Management (PRISM) conceptual framework [31]. A purposive sample of hospital health workers (nurses, midwives and doctors) and EN-BIRTH data collectors was used. Interview audio recordings were transcribed, translated and coded using a priori code and included constructs for Technical, Organisational and Behavioural factors. NVivo 12 software was used to manage data. Respondents also completed a checklist regarding: who usually gives the uterotonic, documents care, which documents uterotonics are recorded in, the order documentation occurs, and estimations of how long after birth uterotonics are documented. More information is available within this supplement [32].

## Results

Across five study hospitals, 23,724 (99.6%) women consented to participate, with 23,015 (97.0%) observed and 20,632 (86.6%) completing an exit survey. Register extraction was completed for 22,002 (92.7%) women (Fig. 2). Participant characteristics are shown in Table 1. Nearly half of participants were presenting with their first pregnancy and participants from Tanzania were most likely to be multiparous (2+ previous births). The proportion of normal vaginal births varied between hospitals, from 26.4% in Azimpur, BD to 91.6% in Temeke, TZ (Table 1). The highest proportion of caesarean births were in Azimpur BD (72.8%) and Muhimbili, TZ (55.8%). 688 (3.2%) women experienced PPH during the study.

**Table 1:** Characteristics of women observed in labour and delivery wards, EN-BIRTH study (*n* = 23,015)

	Hospitals					Total
	Bangladesh		Nepal	Tanzania		
	Azimpur Tertiary	Kushtia District	Pokhara Regional	Temeke Regional	Muhimbili National	
	n (%)	n (%)	n (%)	n (%)	n (%)	
Total	**2910**	**2412**	**7370**	**6748**	**3575**	**23,015**
**Woman’s Age**						
< 18 years	25 (0.9)	3 (0.1)	311 (4.2)	26 (0.4)	8 (0.2)	373 (1.6)
18–19 years	475 (16.3)	197 (8.2)	817 (11.1)	767 (11.4)	159 (4.4)	2415 (10.5)
20–24 years	1158 (39.8)	954 (39.6)	3080 (41.8)	2314 (34.3)	722 (20.2)	8228 (35.8)
25–29 years	867 (29.8)	736 (30.5)	2114 (28.7)	1697 (25.1)	1134 (31.7)	6548 (28.5)
30–34 years	297 (10.2)	373 (15.5)	827 (11.2)	1146 (17)	924 (25.8)	3567 (15.5)
35+ years	88 (3)	149 (6.2)	221 (3)	798 (11.8)	628 (17.6)	1884 (8.2)
**Woman’s education**						
No education	39 (1.3)	77 (3.2)	268 (3.6)	202 (3)	66 (1.8)	652 (2.8)
Primary incomplete	111 (3.8)	127 (5.3)	252 (3.4)	81 (1.2)	45 (1.3)	616 (2.7)
Primary complete	339 (11.6)	347 (14.4)	302 (4.1)	31 (0.5)	5 (0.1)	1024 (4.4)
Secondary incomplete	985 (33.8)	954 (39.6)	1637 (22.2)	4053 (60.1)	1299 (36.3)	8928 (38.8)
Secondary complete or higher	1273 (43.7)	870 (36.1)	4509 (61.2)	2346 (34.8)	2146 (60)	11,144 (48.4)
Don’t know	163 (5.6)	37 (1.5)	402 (5.5)	35 (0.5)	14 (0.4)	651 (2.8)
**Parity**						
Nullipara	1350 (46.4)	1038 (43)	4402 (59.7)	2917 (43.2)	1363 (38.1)	11,070 (48.1)
Multipara	1504 (51.7)	1369 (56.8)	2961 (40.2)	3816 (56.6)	2207 (61.8)	11,857 (51.5)
Missing	56 (1.9)	5 (0.2)	7 (0.1)	15 (0.2)	5 (0.2)	88 (0.4)
**Mode of birth**						
Normal vaginal birth	767 (26.4)	1364 (56.6)	5840 (79.2)	6184 (91.6)	1506 (42.1)	15,661 (68)
Vaginal births: Breech, Vacuum/Forceps	1 (0)	0 (0)	349 (4.8)	10 (0.1)	9 (0.2)	369 (1.6)
Caesarean Section	2119 (72.8)	972 (40.3)	1140 (15.5)	472 (7.0)	1995 (55.8)	6698 (29.1)
**Estimated Blood Loss at birth**						
Normal: ≤500mls	2792 (97.2)	2236 (95.9)	6993 (95.6)	6289 (96.2)	3026 (90.1)	21,336 (95.2)
PPH: > 500 - ≤1000 mls	48 (1.7)	63 (2.7)	133 (1.8)	157 (2.4)	243 (7.2)	644 (2.9)
Severe PPH > 1000 mls	6 (0.2)	11 (0.5)	3 (0.04)	12 (0.2)	12 (0.4)	44 (0.2)
Missing	26 (0.9)	22 (0.9)	185 (2.5)	80 (1.2)	79 (2.4)	392 (1.8)

### Objective 1: Numerator validation

**Fig. 3:**
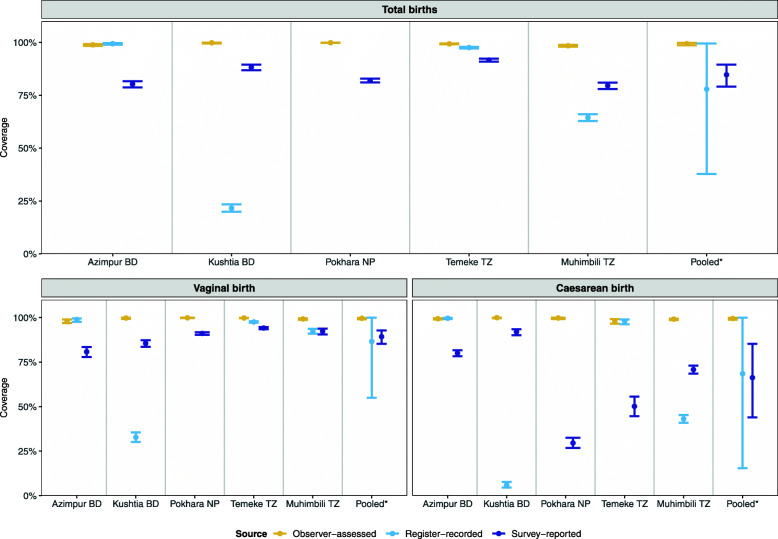
Coverage rates for uterotonic administration measured by observation, register and exit-survey (95% CI), EN-BIRTH study (*n* = 23,015). *n* = 23,015 observer assessed births; 20,632 women with survey-reported births & 14,221 with register records, (noting that for validity analysis, *n* = all register data from Tanzania + revised register data from Bangladesh) [28]. BD = Bangladesh, NP = Nepal, TZ = Tanzania. Pokhara, Nepal has no register column for recording uterotonics (*n* = 6838). *Pooled using random effects

Observed uterotonic coverage was consistently high across all sites and modes of birth (range from 98.4% in Muhimbili, TZ to 99.9% in Pokhara, NP) (Fig. 3). Of those administered uterotonics, > 99% received oxytocin, irrespective of mode of birth (Additional file 8).

#### Exit-interview survey-reported findings

Survey-reported uterotonic coverage ranged from 79.5% in Muhimbili to 91.7% in Temeke TZ; 84.7% (95% CI 79.1–89.5) overall (Additional file 9). Women who had a vaginal birth were more likely to accurately report receiving uterotonics compared with women who gave birth by caesarean (Fig. 3). Survey-reported coverage for vaginal births was 89.3% (96% CI 85.3–92.8) overall and ranged from 80.8% in Azimpur BD to 94.1% in Temeke TZ. For caesarean births survey-reported coverage was 66.3% (95% CI 44.0–85.3) and ranged from 50.2% in Temeke TZ to 92% in Kushtia BD (Additional file 9). The largest differential between survey-reported uterotonic coverage was in Pokhara NP where observer-assessed coverage was 99.9% (95% CI 99.8–100%) compared with 91.1% (95% CI 90.4–91.8) survey-reported for vaginal births, and 29.6% (95% CI 26.8–32.5) survey-reported for caesarean births (Additional file 9).

**Fig. 4:**
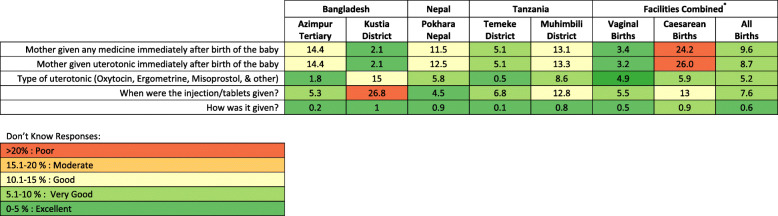
‘Don’t Know’ replies for exit-survey reported uterotonic provision, EN-BIRTH study (*n* = 20,632) *individually weighted mean. Cut-off ranges adapted from WHO Data Quality Review, Module 2 "Desk review of data quality” [33]

Women who had a caesarean section were more likely to report “don’t know” for any uterotonic indicator than those with vaginal birth. “Don’t know” replies were highest (> 20%) for women with caesarean births reporting on medication administration immediately after birth (Fig. 4).

Descriptive analysis comparing reported coverage of potential combined uterotonic indicator options with observed coverage, showed no difference between the various combinations (Additional file 7).

#### Register-recorded findings

**Fig. 5:**
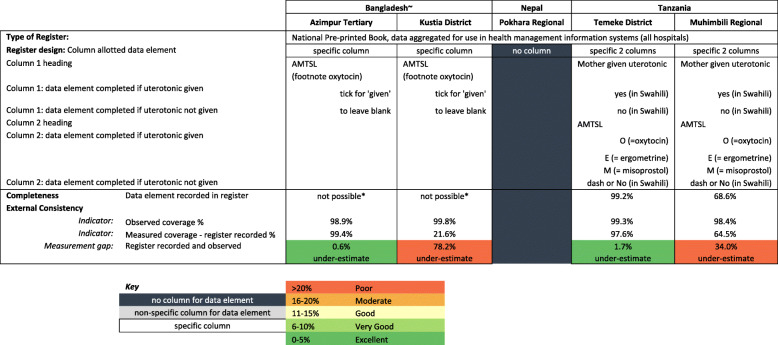
Hospital register design and completion for uterotonics by site, EN-BIRTH study (*n* = 14,211). Register Recorded *n* = 14,221 (for validity analysis *n* = all register data from Tanzania + revised register data from Bangladesh) [28]. ~ Revised Register design, further details available in Additional file 4. *Completeness calculations are “not possible” for Bangladesh registers as the instructions state leave blank if intervention/practice is not done. Cut-off ranges adapted from WHO Data Quality Review, Module 2 “Desk review of data quality” [33]

For hospitals with a specific column, register-recorded uterotonic coverage was 77.9% (95% CI 37.8–99.5) and ranged from 21.6% (Kushtia, BD) to 99.4% (Azimpur, BD). Register-recorded coverage was lowest in Pokhara NP where this data element is not captured (Fig. 5 and Additional file 10). When capturing uterotonics, register-recorded coverage estimates were higher for vaginal births (86.6, 95% CI 55.0–100.0) than caesareans (68.5, 95% CI 15.5–100.0).

Percent agreement between register-recorded and observer-assessed coverage was higher with not recorded results excluded: 86.1% (95% CI 48.5–100.0) for all modes of birth combined, compared to 77.2% (95% CI 37.7–99.3) when not-recorded results were included as ‘not given’ (Additional file 10). Positive predictive value was > 99% for all modes of birth (Additional file 10).

Descriptive analysis of the Bangladesh specific results found that register-recorded coverage of uterotonic administration increased with the introduction of revised registers that included a specific column for third stage labour management. In Azimpur there was an 81.6% increase in the number of register-recorded cases, and 21.6% increase in Kushtia (Additional file 11).

### Objective 2: Denominator comparison

Uterotonic coverage was over-estimated using the live birth denominator in all EN-BIRTH hospitals, the absolute difference ranged between − 1.3 and − 6.8%, and relative difference ranged from − 0.1 to 0 (Table 2).

**Table 2:** Denominator comparisons for uterotonic indicator, EN-BIRTH study (*n* = 23,015)

	Bangladesh		Nepal	Tanzania	
	Azimpur Tertiary	Kushtia District	Pokhara Regional	Temeke Regional	Muhimbili National
Number of women who gave birth	2910	2412	7370	6748	3575
Uterotonic observed given	2858	2333	7221	6653	3485
Total births	2936	2459	7442	6869	3765
Live births	2896	2308	7175	6634	3509
Uterotonic coverage among women who gave birth (%)	98.9	99.8	99.9	99.3	98.4
Uterotonic coverage using live birth denominator (%)	98.7	101.1	100.6	100.3	99.3
Uterotonic coverage using all birth denominator (%)	97.3	94.9	97.0	96.9	92.6
Relative difference %	0.0	−0.1	0.0	0.0	−0.1
Absolute difference %	−1.3	−6.2	−3.6	−3.4	−6.8

*Legend: N* = 23,051 women observed to give birth

Uterotonic coverage is calculated using number of women who gave birth (rather than “all” or “live” births)

### Objective 3: Gaps analysis for coverage and quality of care, and measurement

**Fig. 6:**
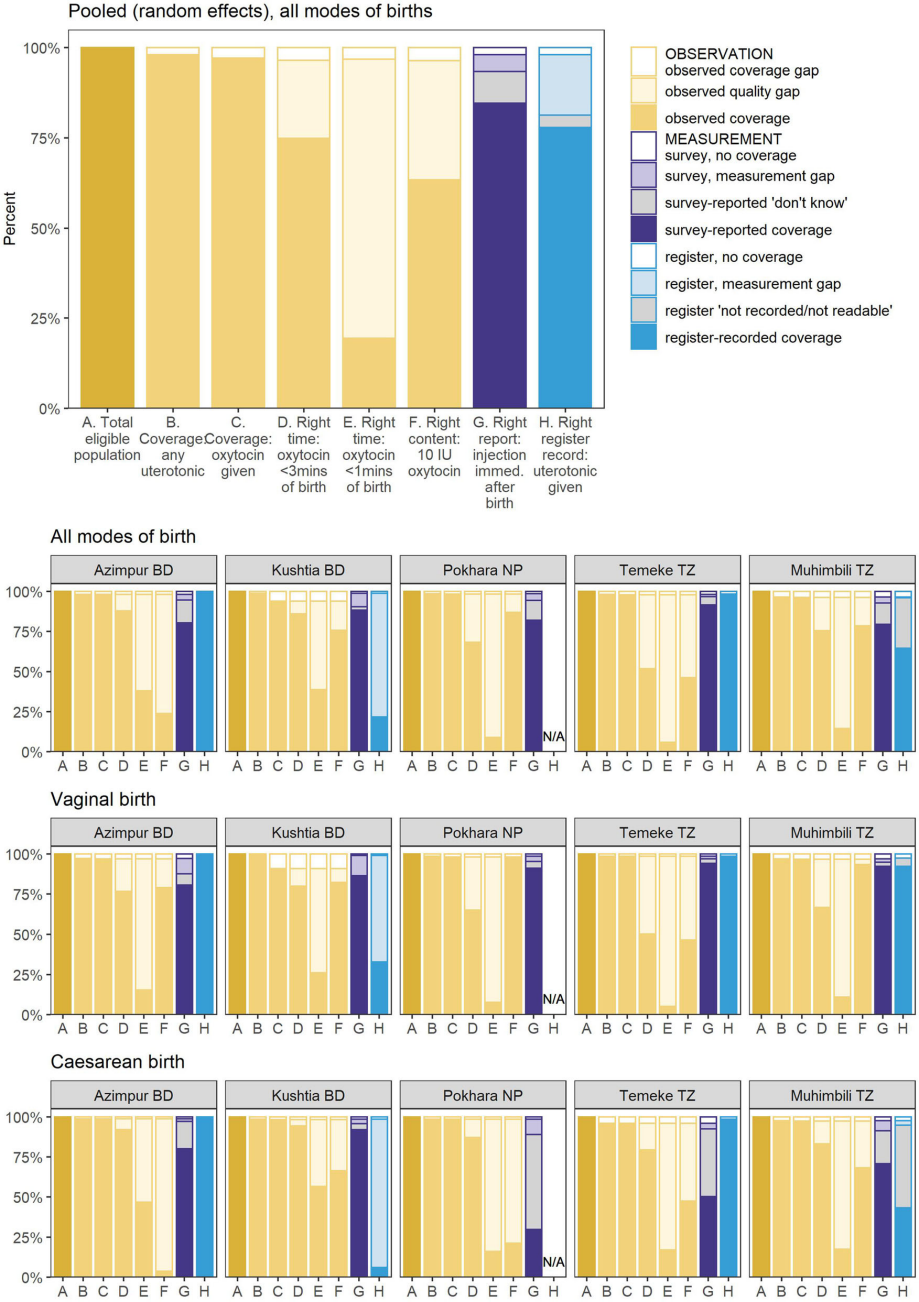
Gap analysis for uterotonic coverage and quality, EN-BIRTH study. *N* = 23,015 observer assessed births: 20,632 survey reported births and 14,221 register recorded (all cases in Tanzania and those from revised register data from Bangladesh) BD = Bangladesh, NP = Nepal, TZ = Tanzania

**Fig. 7:**
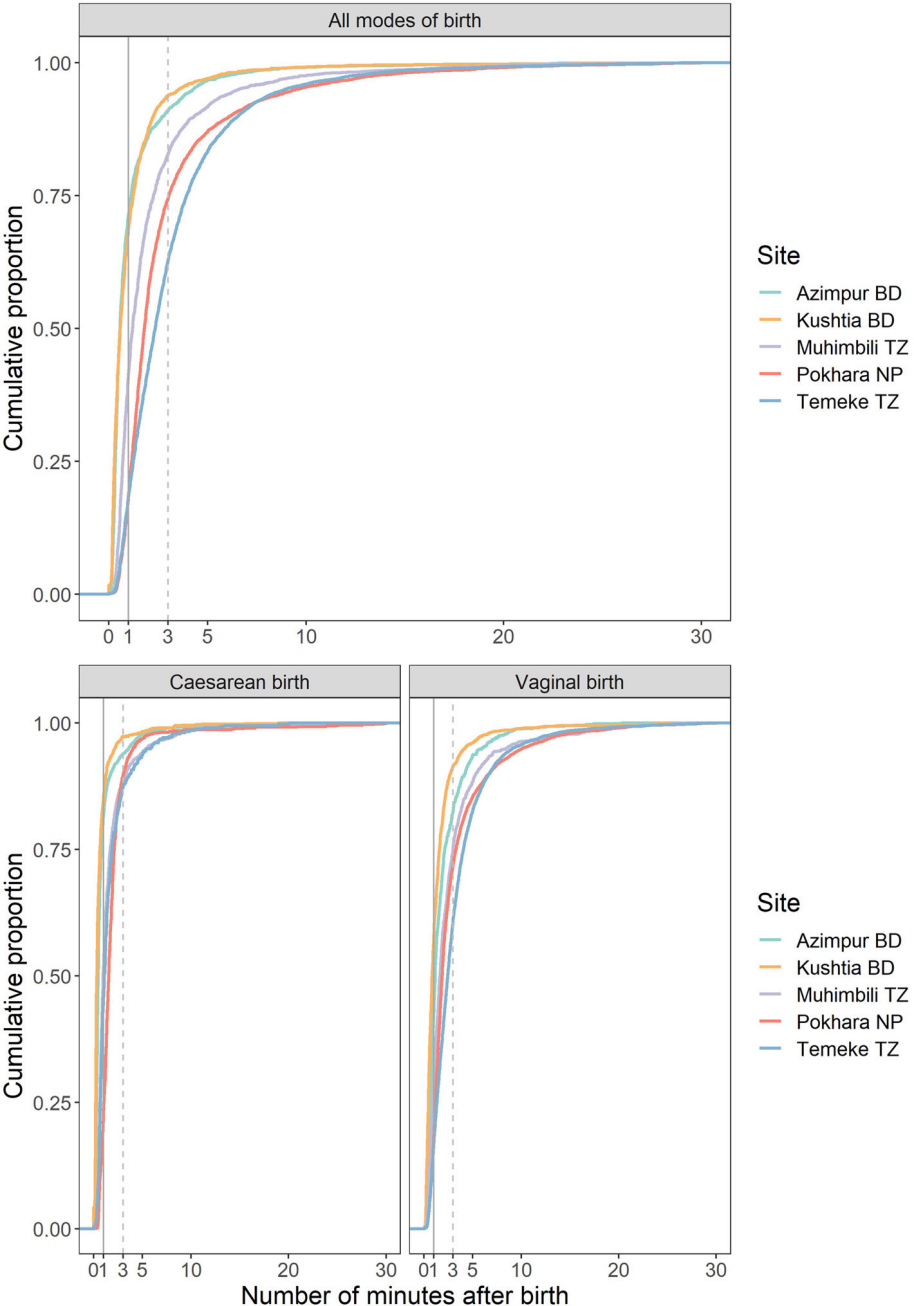
Timing of oxytocin administration, EN-BIRTH study (*n* = 22,121). *N* = 22,121 (women observer-assessed to receive oxytocin from 0 to 30 min after birth). BD = Bangladesh, NP = Nepal, TZ = Tanzania

The coverage gap for oxytocin for PPH prevention within 30 min of birth was small (1.9%) in all sites (Fig. 6). Quality gap analysis showed timing distribution was different between each hospital and by mode of birth (Additional file 12). Oxytocin was administered more quickly for caesarean births than vaginal births, and overall most women (88.8% Azimpur, 90.3% Kushtia, 68.6% Pokhara, 52.4% Temeke and 76.7% Muhimbili) received oxytocin within 3 min (the “right time”, Fig. 7). The distribution of Oxytocin dose, “right content”, showed that 66.3% of women received 10 IU of Oxytocin, 21.8% 20 IU, and 4.25% 40 IU (Additional file 13). Of those who received 40 IU, 2.2% were observed to have a blood loss of > 500mls (Additional file 14). Women giving birth via caesarean section were more likely to receive higher doses of Oxytocin than those with vaginal births. In observed cases, the route of administration was intramuscular (IM) for 65.2%, and intravenous (IV) in 34.3% of births (Additional file 8).

The measurement gap was 18.1% for register-recorded and 6% for survey-reported coverage. For women who had a vaginal birth, 39% (ranging from 0.7% in Azimpur to 67.6% at Temeke) could report the purpose of the uterotonic medication (‘to prevent haemorrhage’). For caesarean births, this dropped to 6.9% (ranging from 0.3% in Azimpur to 17.1% in Temeke) (Additional file 8). Less than 2.5% of women could name the drug they were given (Additional file 8).

### Objective 4: Barriers and enablers to data collection

**Fig. 8:**
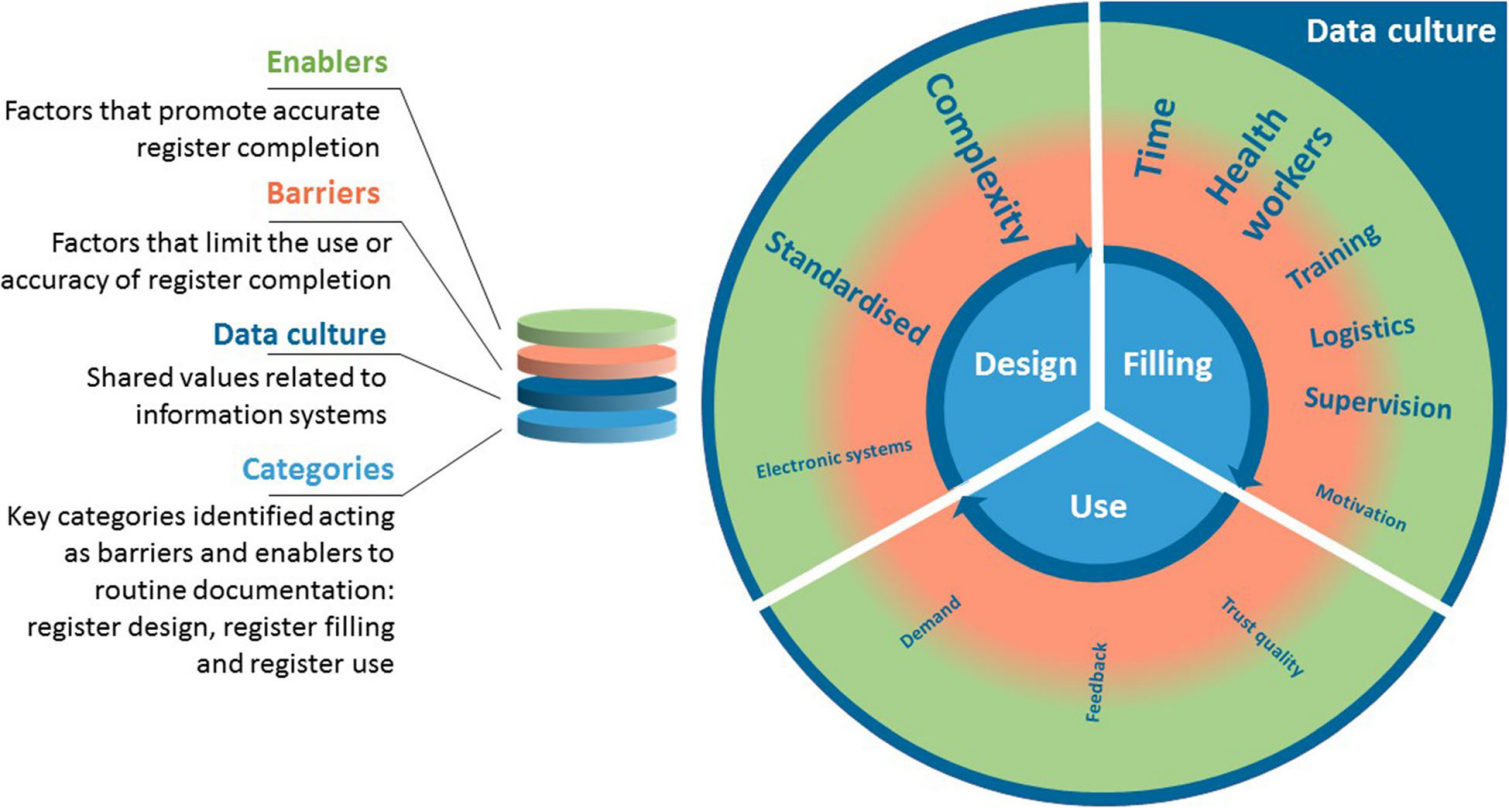
Barriers and enablers to routine register recording of uterotonic administration, EN-BIRTH study. This Figure illustrates the overall barriers and enablers to facility based data collection identified by EN-BIRTH participants [32]. The bold text are the issues specific to uterotonics administration. The transition from red to green is a reminder that most factors identified by participants could serve as either a barrier or enabling factor depending on the facility-level resources and management

We identified three categories under which to group emerging themes regarding barriers and enablers to routine recording of uterotonic administration in hospital registers: 1) Register or system design; 2) Register filling or completion; 3) Register use (Fig. 8) [32].

#### Register or system design

Within this category, two themes emerged for uterotonic recording. Focus group participants talked about the complexity of health data systems and the specific register design for uterotonics. Across all sites, health workers identified multiple places where they were expected to document information about care during the third stage of labour, including the register, clinical records, partograph, and drug chart. Many staff reported they did not know who would be taking primary responsibility for documentation (Additional files 15 and 16).

These challenges were underlined in Kushtia BD and Muhimbili TZ, where register performance was lower:

*‘She will go to the nursing station to do her documentation in the health management system tool, then fills the midwifery book, the books are in different places and are far from the patient and the delivery room.’*

–Health worker, Muhimbili TZ

Participants reported that design of the register, amount of space and inclusion of a specific column for the uterotonic documentation is needed to facilitate high quality data collection:

*‘There is no such space to record, maybe we have administered a certain amount of oxytocin or ergometrine, no space for that.’*

–Data collector, Muhimbili TZ

#### Register filling or completion

Lack of health workers (quantity and capacity) was identified as a critical challenge throughout all the focus group discussions and was a key barrier to uterotonic data collection among other indicators:

*‘We have a shortage of manpower and time … We need time to examine and provide the treatment thoroughly.... But also we have to maintain the documentation.’*

–Health worker, Azimpur BD

Evidence from Temeke TZ suggests that some of these challenges can be addressed with good organisation of workspaces to ensure that clinical environments are enabling with the required register books, computers, and stationary positioned in convenient clinical locations that facilitate health workers to remain near service users:

*‘There is a specific place kept and arranged for documenting all the provided care … they are supposed to be there, equipment like books for recording and pens [are there], and it is not far’*

–Data collector, Temeke TZ

Healthcare staff reported that they are usually completing care during the third stage of labour and documentation simultaneously. Staff from Kushtia and Muhimbili identified the location of registers as problematic, which was also identified as a register-recording barrier, across all sites, for women giving birth in the operating theatres Fig. 8.

Participants from Kushtia BD and Muhimbili TZ reported supply challenges with basic equipment including multiple register stock-outs in Kushtia, and the requirement for staff to supply their own pens:

*‘We usually buy our pen ourselves, we do not get a pen from the office.’*

-Health worker, Kushtia BD

#### Register use

Respondents from Temeke TZ and Azimpur BD reported these sites have instituted regular opportunities for staff to use and reflect on their data. Moreover, staff in these hospitals were using data for a variety of purposes in their own practice:

*‘These documents show what the patient is suffering from and what medication is given … Also these documents are important for research works, planning, improving health services, helping secure you in the court, and in statistics. The documents are very important in improving health services.’*

–Data collector, Temeke TZ

Feedback was consistently valued by staff in all hospitals, and health workers suggested leadership was an enabling factor for documentation. Temeke TZ had highly accurate register reporting for uterotonics. Staff reported being well-supported by management with regular feedback, training and opportunities to use their data during budget planning, stock management, audit and monitoring:

*‘Leadership in general from the lower level to the upper level should have good communication and cooperation to ensure that everything is well documented and records are kept with good quality.’*

–Data collector, Temeke TZ

Staff from EN-BIRTH sites with more accurate register-recording of uterotonic coverage reported training as an essential component. Managerial gaps and lack of training were cited as barriers to documentation in Kushtia, the site with lowest performing register-recording.

‘*However we are not well trained’.*

–Health worker, Kushtia BD

## Discussion

Postpartum haemorrhage remains a leading cause of preventable maternal mortality. Despite WHO recommendations for universal access to prophylactic uterotonics, there are no nationally representative data to track coverage and quality of this intervention [6, 7]. EN-BIRTH is the largest measurement validation study to date, with more than 10 times the number of participants of previous studies, and assessed both survey-reported and register-recorded indicators.

Survey-reported data for assessing uterotonic coverage was problematic, with high “don’t know” replies for caesarean births, and lower accuracy than the better performing registers. Our findings align with previous validation studies suggesting low individual-level accuracy for survey measures of uterotonic coverage [20–24]. There was also poor consistency between observer-assessed and survey-reported events around timing, and type of uterotonic administration. Our survey data was collected at exit-interview on discharge from the hospital; and we anticipate that the discrepancy between woman’s report and gold standard data may increase over time in line with other study findings [20–24].

Our results underline that accurate report in surveys is challenging for events around the time of birth, especially for women receiving more complex clinical care (e.g. PPH management or caesarean section). This is unlikely to be recall alone; the women’s knowledge will depend on the quality of information provided by healthcare staff, and if informed consent was elicited [20, 24]. Indicators regarding knowledge of care and rationale could serve as tracers for respectful care, as women have a right to informed decision making and autonomy [34, 35]. These rights are increasingly recognised: respectful and dignified care was the number one demand from the recent ‘what women want’ campaign with > 1 million participants across 114 countries [36]. Participants experiencing caesareans were less likely to report that the health worker explained the purpose of uterotonic medication (Additional file 8). Given caesarean section rates are increasing globally [37], further research is needed on how accuracy of women’s report is effected by both direct (anaesthetics or sedatives) and indirect processes. This includes what information is given to women about treatment of them and their baby, and issues around gaining her informed consent.

Register completion varies [7, 20, 27, 38–41]. The two highest performing hospitals achieved high sensitivity (97.6–99.5%) and percent agreement (97.3–99.0%) between register-recorded and observer-assessed coverage. Pokhara NP had no column or space available in the register for uterotonic documentation. These findings draw attention to the requirement for clear register design around priority measures and the need for more global guidance and standardisation, especially given there are multiple stakeholders and only limited space and capacity for the inclusion of data elements in routine registers. Wider use of national electronic HMIS tools, such as District Health Information Software 2 (DHIS2) [42, 43], provide important platforms for faster uptake. Evidence from Nigeria suggests that tracking of maternal and newborn indicators through HMIS is possible with strong multi-partner collaboration at all levels of the health system to rationalise data flow, and provide supervision with data quality review, feedback and data reporting [27].

Register design is necessary but not sufficient to achieve high quality data, inclusive training and implementation strategies are also imperative. Despite sharing the same register design and layout, results differed between Temeke and Muhimbili TZ, and between Azimpur and Kushtia BD after implementation of the new national register. Our results support evidence that data collection and management processes represent a heavy workload for health workers [39, 44–46], who face competing priorities and challenges on their time. Managerial support for data collection including supervision, feedback and review are therefore essential [27].

Maternal mortality remains high in many settings despite good coverage of facility births [47]; this divergence in expectation is usually attributed to quality gaps in service provision. Yet to be sure, we need more granular data on the content and quality of care. There was a quality gap for timing with less than 20% of women receiving oxytocin within 1 minute of birth as recommended by WHO [5], although the majority were within ≤3 min (Fig. 7). We recommend further research around the precise timing need for uterotonic administration [48], especially as early indications from an ongoing trial assessing tranexamic acid to treat PPH, suggest that the positive effect of administration reduces with every minute of delay [49].

Uterotonic coverage was high in our study sites, although these high caseload referral centres are not representative of all facilities in LMICs. Several studies indicate that quality of care is lower in primary-level facilities, especially those with a low case-load [47]. We used the elements of timing, and dose of drug use as quality measures. However, Oxytocin is light and heat sensitive and should be stored between 2 and 8 °C for extended shelf life [5]. Stock-outs, poor adherence to manufacturer guidelines and prolonged exposure to high temperatures reduce the availability of effective Oxytocin at the point of care [50]. Oxytocin samples tested from multiple LMICs were found to have insufficient active ingredient, with up to 74% of tested samples failing [51, 52]. Given this would likely fall outside routine measurement systems, further work to examine these aspects of quality are needed.

Denominators are crucial for public health decision-making [53]. Worldwide, four in every five births are estimated to be taking place in facilities and almost 81% are supported by a skilled birth attendant, but the poorest women in the poorest countries are still without access [1, 25]. Whilst most of the numerator of women given injectable uterotonics may be captured in a facility (given this is WHO policy), a denominator of only facility births omits home births [16]. Some countries do have a policy supporting misoprostol use for non-facility births, but these data are not currently being measured. Many LMICs estimate denominators via census-derived population estimates (i.e. for immunisation) [54]. This is also feasible using an estimated total birth denominator for a given population, such as a district. If there are many births in the private sector, HMIS should aim to include the count data of women given uterotonics and the relevant denominator. In India, the private and non-profit sectors are now mandated to report selected data to the government HMIS [54, 55].

### Strengths and limitations

EN-BIRTH study strengths include use of direct observation as gold standard, the large number of participants, time-stamped data, stratification of results by mode of birth, and five differing hospitals from three LMICs. Unfortunately, even the high number of observed births were not able to mitigate statistical challenges validating indicators with high prevalence, especially those only calculated for observations with ≥10 counts in each column of the 2 × 2 tables to assess sensitivity, specificity, inflation factor and area under the curve [56]. The gold standard could also be susceptible to errors in data recording and interpretation, especially for estimated blood loss. Some of these risks were reduced via use of the custom-built tablet-based application, standardised training, and supervision throughout data collection. We also assessed inter-observer error by double entering observations for 5% of cases, and found good agreement for uterotonics (Additional file 6). Study data were collected in CEmOC level hospitals where higher case-loads, access to multidisciplinary teams, and potentially higher levels of supervision and training might mean that both the provision and recording of uterotonic drugs are completed to a higher standard. The Hawthorne effect (whereby a study changes practice) could have resulted in improved register documentation and/or uterotonic provision by health workers. However, comparison of registers pre-study with during-after register records shows no significant change in completeness or documentation practises [28].

### Research gaps for improving measurement

Systematic research and investment in implementation are needed to improve register design and use. Where coverage is high, a simple uterotonics coverage indicator might be insufficient to drive quality improvement. Other measures may be required such as health facility assessments regarding drug quality, and stock management, or use of specific audits. There is potential for linking databases (such as survey and facility-based data) but this may require special studies and complex analyses [6, 57–60].

Assessment of data flow within HMIS and inter-operability with related platforms, such as supply logistics systems, are also needed. This could be undertaken as part of a feasibility assessment of maternal and newborn HMIS tool kits in a range of LMICs and humanitarian settings. It should include data quality assessments at different levels of the HMIS, including costs for data collection and assessment of usefulness to policymakers.

## Conclusions

EN-BIRTH findings for uterotonics measurement are compatible with existing evidence suggesting that asking women about clinical interventions during or immediately after birth is unreliable [20–24], especially following caesarean section. Based on this evidence, we do not recommend the addition of a uterotonic indicator to household survey platforms such as DHS and MICS. Registers have potential to accurately capture coverage of uterotonics and could provide timely data; however, this requires work on register design, standardisation and improved global guidance. A well-designed, parsimonious, standardised register is necessary but not sufficient to collecting consistent high-quality data. Importantly, those who enter the data are often over-worked health professionals who need to know why these data matter for their own use, and for the women they care for. Feedback mechanisms and data use are important enablers to drive improvements in register-recording practices.

## Supplementary Information

**Additional file 1.** Summary of previous validation for measures of uterotonic administration.

**Additional file 2.** Ethical approval of local institutional review boards, EN-BIRTH study.

**Additional file 3.** STROBE Checklist.

**Additional file 4.** Data collection dates by site, EN-BIRTH study.

**Additional file 5.** Facility register design and completion approaches for uterotonics by site, EN-BIRTH study (*n* = 22,002).

**Additional file 6.** Inter-observer agreement for uterotonic administration using Kappa, EN-BIRTH study.

**Additional file 7.** Survey- reported uterotonic indicator combinations compared with observer-assessed coverage, EN-BIRTH study.

**Additional file 8.** Descriptive uterotonic coverage data: observer-assessed, exit-survey reported and register-recorded findings, EN-BIRTH study (*n* = 23,015).

**Additional file 9.** Individual-level validation of exit-survey report for uterotonic administration, EN-BIRTH Study (*n* = 23,051).

**Additional file 10.** Individual-level validation of register recording for uterotonic administration, EN-BIRTH study (*n* = 15,645).

**Additional file 11.** Comparison of uterotonic coverage measurement using original and revised Bangladesh registers, EN-BIRTH study (*n* = 5207).

**Additional file 12.** Association testing for timing of Oxytocin administration, EN-BIRTH Study (*n* = 22,121).

**Additional file 13.** Oxytocin dose by EN-BIRTH site and mode of birth, EN-BIRTH study (*n* = 22,269).

**Additional file 14.** Estimated Blood Loss (EBL) compared with Oxytocin coverage, EN-BIRTH Study.

**Additional file 15.** Assessment of routine recording responsibilities for uterotonic provision, EN-BIRTH Study.

**Additional file 16.** Register recording order and prioritisation for uterotonic provision, EN-BIRTH study.

## Data Availability

The datasets generated during and/or analysed during the current study are available on LSHTM Data Compass repository, https://datacompass.lshtm.ac.uk/955/.

## Abbreviations

AMTSL: Active management of the third stage of labour
BD: Bangladesh
CEmOC: Comprehensive emergency obstetric care
CIFF: Children’s Investment Fund Foundation
DHS: Demographic and Health Surveys Program
DHIS2: District Health Information Software 2
EN-BIRTH: *Every Newborn*-Birth Indicators Research Tracking in Hospitals study
EPMM: Ending Preventable Maternal Mortality
HMIS: Health Management Information Systems
icddr,b: International Centre for Diarrheal Disease Research, Bangladesh
IHI: Ifakara Health Institute
IM: Intramuscular
IU: International units
LMIC: Low and Middle Income Country
LSHTM: London School of Hygiene & Tropical Medicine
MUHAS: Muhimbili University of Health and Allied Sciences
MICS: Multiple Indicator Cluster Survey
NP: Nepal
PPH: Postpartum Haemorrhage
PRISM: Performance of Routine Information System Management
TZ: Tanzania
WHO: World Health Organization
