# Supplementary material for: Uterotonics for prevention of postpartum haemorrhage: EN-BIRTH multi-country validation study
Source: BMC Pregnancy Childbirth. 2021 Mar 26;21(Suppl 1):230. doi: 10.1186/s12884-020-03420-x (PMC7995712; doi:10.1186/s12884-020-03420-x)
Supplement: Supplementary file 1 — Additional file 1. Summary of previous validation for measures of uterotonic administration. [file 12884_2020_3420_MOESM1_ESM.pdf]

Every Newborn BIRTH multi-country validation study: informing measurement of coverage and quality of maternal and newborn care

Uterotonics for prevention of postpartum haemorrhage: EN-BIRTH multi-country validation study

Additional File 1: Summary of previous validation for measures of uterotonic administration

	Sample	Maternal Exit Survey*	% Don't Knows	Follow up Survey	% Don't Knows	Maternity Register
EN-BIRTH (2019), Bangladesh, Nepal, Tanzania	23,015 Observed 20632 Maternal Exit	IF	9.60%			<5 cell count 96% agreement
Bhattacharya (2019) Nigeria [1]	1867 Exit 442 FU	IF	0%	IF	1%	<5 cell count 93% agreement
Blanc (2016) Kenya [2]	552	<5 cell count	<5%			
Blanc (2016) Mexico [3,	592	IF	38.30%			
McCarthy (2016) Kenya [4]	445 Exit 437 FU	Sample too small	4.50%	Sample too small	8.50%	Not done
Stanton (2013) Mozambique [5]	289	Neither AUC or IF	Not reported			Not done
Broughton (2013) Afghanistan [6]	600					IF not reported, AUC <0.6 (below threshold)

*AUC (area under the curve) defined as ≥0.6, IF 0.75-1.25.

References

1. Bhattacharya AA, Allen E, Umar N, Usman AU, Felix H, Audu A, Schellenberg JR, Marchant T: **Monitoring childbirth care in primary health facilities: a validity study in Gombe State, northeastern Nigeria**. *Journal of global health* 2019, **9**(2).
2. Blanc AK, Diaz C, McCarthy KJ, Berdichevsky K: **Measuring progress in maternal and newborn health care in Mexico: validating indicators of health system contact and quality of care**. *BMC pregnancy and childbirth* 2016, **16**(1):255.
3. Blanc AK, Warren C, McCarthy KJ, Kimani J, Ndwiga C, RamaRao S: **Assessing the validity of indicators of the quality of maternal and newborn health care in Kenya**. *Journal of global health* 2016, **6**(1).
4. Broughton EI, Ikram AN, Sahak I: **How accurate are medical record data in Afghanistan's maternal health facilities? An observational validity study**. *BMJ open* 2013, **3**(4):e002554.
5. McCarthy KJ, Blanc AK, Warren CE, Kimani J, Mdawida B, Ndwidga C: **Can surveys of women accurately track indicators of maternal and newborn care? A validity and reliability study in Kenya**. *Journal of global health* 2016, **6**(2).
6. Stanton CK, Rawlins B, Drake M, dos Anjos M, Cantor D, Chongo L, Chavane L, da Luz Vaz M, Ricca J: **Measuring coverage in MNCH: Testing the validity of women's self-report of key maternal and newborn health Interventions during the peripartum period in Mozambique**. *PLoS One* 2013, **8**(5):e60694.
