# Supplementary material for: Uterotonics for prevention of postpartum haemorrhage: EN-BIRTH multi-country validation study
Source: BMC Pregnancy Childbirth. 2021 Mar 26;21(Suppl 1):230. doi: 10.1186/s12884-020-03420-x (PMC7995712; doi:10.1186/s12884-020-03420-x)
Supplement: Supplementary file 4 — Additional file 4. Data collection dates by site, EN-BIRTH study. [file 12884_2020_3420_MOESM4_ESM.pdf]

Every Newborn BIRTH multi-country validation study: informing measurement of coverage and quality of maternal and newborn care

Uterotonics for prevention of postpartum haemorrhage: EN-BIRTH multi-country validation study

Additional File 4: Data collection dates by site, EN-BIRTH study.

	Bangladesh		Nepal	Tanzania		
**EN-BIRTH study**	**Azimpur Tertiary**	**Kushtia District**	**Pokhara Regional**	**Temeke Regional**	**Muhimbili National**	**All sites**
**Tablet data collection dates**	17/8/17 to 30/4/18	11/7/17 to 30/5/18	17/7/17 to 31/7/18	3/7/17 to 30/5/18	3/7/17 to 28/2/18	
Duration	8 months	10 months	1 year	10 months	7 months	
Original register used:	17/8/17 to 18/10/17	25/8/17 to 27/9/17 (due to short supply)	Not applicable	Not applicable	Not applicable	
Revised register used:	19/10/17 to 30/4/18	11/7/17 to 24/8/17 and 28/9/17 to 30/5/18	Not applicable	Not applicable	Not applicable	
**Register extraction comparison dates to assess biases**						
**Pre-study**	1/1/16 to 31/12/16	1/1/16 to 31/12/16	1/4/16 to 31/3/17	1/1/16 to 31/12/16	1/1/16 to 31/12/16	
Duration	12 months	12 months	12 months	12 months	12 months	
**During/after-study**	17/8/17 to 17/8/18	11/7/17 to 11/7/18	17/7/17 to 17/7/18	3/7/17 to 3/7/18	3/7/17 to 3/7/18	
Duration	12 months	12 months	12 months	12 months	12 months	
**Time elapsed between delivery and exit survey interview/ days**	n (%)	n (%)	n (%)	n (%)	n (%)	n (%)
**Total**	2844	2331	6922	5752	2783	20632
0-1 day	725 (25.5)	1345 (57.7)	5854 (84.6)	5433 (94.5)	1009 (36.3)	14366 (69.6)
2-3 days	511 (18)	846 (36.3)	833 (12)	181 (3.1)	1098 (39.5)	3469 (16.8)
4+ days	1599 (56.2)	127 (5.4)	154 (2.2)	43 (0.7)	597 (21.5)	2520 (12.2)
Missing	9 (0.3)	13 (0.6)	81 (1.2)	95 (1.7)	79 (2.8)	277 (1.3)
Mean	3.1	1.3	0.6	0.7	3.2	1.4
Median	4.0	0.0	0.0	0.0	2.0	1.0

Sample size was calculated to observe at least 106 observations per intervention per country, based on estimated coverage of intervention during formative research [1].

1. Day LT, Ruysen H, Gordeev VS, et al. "Every Newborn-BIRTH" protocol: observational study validating indicators for coverage and quality of maternal and newborn health care in Bangladesh, Nepal and Tanzania. *Journal of Global Health* 2019; **9**(1).
