# Supplementary material for: Uterotonics for prevention of postpartum haemorrhage: EN-BIRTH multi-country validation study
Source: BMC Pregnancy Childbirth. 2021 Mar 26;21(Suppl 1):230. doi: 10.1186/s12884-020-03420-x (PMC7995712; doi:10.1186/s12884-020-03420-x)
Supplement: Supplementary file 5 — Additional file 5. Facility register design and completion approaches for uterotonics by site, EN-BIRTH study (n = 22,002). [file 12884_2020_3420_MOESM5_ESM.pdf]

Every Newborn BIRTH multi-country validation study: informing measurement of coverage and quality of maternal and newborn care

Uterotonics for prevention of postpartum haemorrhage: EN-BIRTH multi-country validation study

Additional File 5: Facility register design and completion approaches for uterotonics by site, EN-BIRTH study (n=22,002)

	Bangladesh				Nepal	Tanzania	
	Azimpur Tertiary		Kushtia District		Pokhara Regional	Temeke Regional	Muhimbili National
	Original register	Revised register	Original register	Revised register			
**Register design:** Column allotted data element	non-specific	specific column	non-specific	specific column	no column	specific 2 columns	specific 2 columns
Column 1 heading	Drugs given	AMTSL (footnote oxytocin)	Drugs given	AMTSL (footnote oxytocin)		Mother given uterotonic	Mother given uterotonic
Column 1: data element completed if uterotonic given	"NVD drugs given"	tick for 'given'	drug name	tick for 'given'		yes (in Swahili)	yes (in Swahili)
Column 1: data element completed if uterotonic not given	to leave blank	to leave blank	to leave blank	to leave blank		no (in Swahili)	no (in Swahili)
Column 2 heading						AMTSL	AMTSL
Column 2: data element completed if uterotonic given						O (=oxytocin)<br>E (= ergometrine)<br>M (= misoprostol)	O (=oxytocin)<br>E (= ergometrine)<br>M (= misoprostol)
Column 2: data element completed if uterotonic not given						dash or No (in Swahili)	dash or No (in Swahili)
**Completeness** Data element recorded in register	not possible*	not possible*	not possible*	not possible*		99.2%	68.6%
**External Consistency**							
Indicator: Observed coverage %	98.9%	98.9%	99.8%	99.8%		99.3%	98.4%
Indicator: Measured coverage - register recorded %	17.8%	99.4%	0.0%	21.6%		97.6%	64.5%
Measurement gap: Register recorded and observed	81.1% Under-estimate	0.6% Under-estimate	99.8% Under-estimate	78.2% Under-estimate		1.7% Under-estimate	34.0% Under-estimate

Key		
	>20%	Poor
no column for data element	16-20%	Moderate
non-specific column for data element	11-15%	Good
specific column	6-10%	Very Good
	0-5%	Excellent

N=22,002 women with register recorded birth record

For validity analysis, register recorded n=14,221 (all register data from Tanzania + revised register data from Bangladesh) [1]

*Completeness calculations are "not possible" for Bangladesh registers as the instructions state leave blank if intervention/practice is not done.

Cut-off ranges adapted from WHO Data Quality Review, Module 2 "Desk review of data quality" [2]

References

1. Day L, Rahman QS, Rahman A, Salim N, KC A, Ruysen H, Tahsina T, Masanja H, Basnet O, Gore-langton G *et al*: **Assessment of the validity of the measurement of newborn and maternal health-care coverage in hospitals (EN-BIRTH): a mixed-methods observational study** *Lancet Global* (2020) DOI: 10.1016/S2214-109X(20)30504-0.
2. World Health Organisation: **Data quality review: a toolkit for facility data quality assessment. Module 2: Desk review of data quality** WHO. Geneva; 2017.
