# Supplementary material for: Uterotonics for prevention of postpartum haemorrhage: EN-BIRTH multi-country validation study
Source: BMC Pregnancy Childbirth. 2021 Mar 26;21(Suppl 1):230. doi: 10.1186/s12884-020-03420-x (PMC7995712; doi:10.1186/s12884-020-03420-x)
Supplement: Supplementary file 6 — Additional file 6. Inter-observer agreement for uterotonic administration using Kappa, EN-BIRTH study. [file 12884_2020_3420_MOESM6_ESM.pdf]

Every Newborn BIRTH multi-country validation study: informing measurement of coverage and quality of maternal and newborn care

Uterotonics for prevention of postpartum haemorrhage: EN-BIRTH multi-country validation study

Additional File 6: Inter-observer agreement for uterotonic administration using Kappa, EN-BIRTH study

		**Bangladesh**		**Nepal**	**Tanzania**	
	**n**	**Azimpur Tertiary**	**Kushtia District**	**Pokhara Regional**	**Temeke Regional**	**Muhimbili National**
Observation	2544	1.000	1.000	1.000	0.599	1.000
Data Extraction	2131	1.000	0.652	[redacted]	0.060	-0.109

L&D= Labour and delivery
Kappa agreement cut offs for high/ substantial disagreement: ≥0.71 for observation and ≥0.91 for data extraction [1]

Reference

1. Day L, Rahman QS, Rahman A, Salim N, KC A, Ruysen H, Tahsina T, Masanja H, Basnet O, Gore-langton G *et al*: **Assessment of the validity of the measurement of newborn and maternal health-care coverage in hospitals (EN-BIRTH): a mixed-methods observational study** Lancet Global (2020) DOI: 10.1016/S2214-109X(20)30504-0.
