# Supplementary material for: Uterotonics for prevention of postpartum haemorrhage: EN-BIRTH multi-country validation study
Source: BMC Pregnancy Childbirth. 2021 Mar 26;21(Suppl 1):230. doi: 10.1186/s12884-020-03420-x (PMC7995712; doi:10.1186/s12884-020-03420-x)
Supplement: Supplementary file 9 — Additional file 9. Individual-level validation of exit-survey report for uterotonic administration, EN-BIRTH Study (n = 23,051). [file 12884_2020_3420_MOESM9_ESM.pdf]

Every Newborn BIRTH multi-country validation study: informing measurement of coverage and quality of maternal and newborn care

# Uterotonics for prevention of postpartum haemorrhage: EN-BIRTH multi-country validation study

Additional File 9: Individual-level validation of exit-survey report for uterotonic administration, EN-BIRTH Study (n=23,015)

| | **Bangladesh** | | | | **Nepal** | | **Tanzania** | | | | **All sites Pooled** | |
|---|---|---|---|---|---|---|---|---|---|---|---|---|
| | **Azimpur Tertiary** | | **Kustia District** | | **Pokhara Regional** | | **Temeke Regional** | | **Muhimbili National** | | **Random Effects** | |
| | **%** | **95% CI** | **%** | **95% CI** | **%** | **95% CI** | **%** | **95% CI** | **%** | **95% CI** | **%** | **95% CI** |
| **All modes of Birth** | | | | | | | | | | | | |
| Observer-assessed coverage % | 98.9 | (98.4,99.2) | 99.8 | (99.5,99.9) | 99.9 | (99.8,99.9) | 99.3 | (99.1,99.5) | 98.4 | (98,98.8) | 99.4 | (98.7,99.8) |
| Survey reported prevalence % | 80.3 | (78.8,81.7) | 88.2 | (86.8,89.5) | 82.0 | (81,82.8) | 91.7 | (90.9,92.4) | 79.5 | (78,81) | 84.7 | (79.1,89.5) |
| "Don't know" responses % | 14.4 | | 2.1 | | 12.5 | | 5.1 | | 13.3 | | 8.7 | (4.5,14.1) |
| **Current survey - Count "don't know" as "no"** | | | | | | | | | | | | |
| > 10 counts in either column of 2x2 table | Yes | | No | | No | | Yes | | Yes | | | |
| Percent agreement (TN+TP/n) all observed % | 79.8 | | 88.1 | | 83.0 | | 92.0 | | 79.2 | | 84.7 | (79.4,89.4) |
| Sensitivity % (95% CI) | 80.3 | (78.8,81.8) | + | + | + | + | 91.8 | (91,92.5) | 79.7 | (78.1,81.2) | 84.9 | (79.6,89.6) |
| Specificity % (95% CI) | 28.0 | (12.1,49.4) | + | + | + | + | 33.3 | (14.6,57) | 41.7 | (22.1,63.4) | 32.5 | (21.2,44.6) |
| Positive Predictive Value % (95% CI) | 99.2 | (98.8,99.5) | + | + | + | + | 99.7 | (99.6,99.9) | 99.3 | (98.9,99.6) | | |
| Negative Predictive Value % (95% CI) | 1.2 | (0.5,2.6) | + | + | + | + | 1.5 | (0.6,3) | 1.8 | (0.9,3.3) | | |
| AUC and "accuracy" | 0.5 | low | + | + | + | + | 0.6 | Moderate | 0.6 | Moderate | | |
| Inflation Factor and "Bias" | 0.8 | low | + | + | + | + | 0.9 | low | 0.8 | low | | |
| **Consider only "yes" and "no" (exclude don't know)** | | | | | | | | | | | | |
| > 10 counts in either column of 2x2 table | No | | No | | No | | Yes | | Yes | | | |
| Percent agreement (TN+TP/n) 2x2 table only % | 93.1 | | 90.1 | | 93.8 | | 96.4 | | 91.3 | | 93.1 | (90.7,95.2) |
| Sensitivity (95% CI) | 93.8 | (92.8,94.8) | + | + | + | + | 96.6 | (96.1,97.1) | 91.8 | (90.6,92.9) | 93.5 | (91.1,95.5) |
| Specificity (95% CI) | 18.2 | (5.2,40.3) | + | + | + | + | 17.6 | (3.8,43.4) | 26.3 | (9.1,51.2) | 17.3 | (7.4,29.4) |
| Positive Predictive Value (95% CI) | 99.2 | (98.8,99.5) | + | + | + | + | 99.7 | (99.6,99.9) | 99.3 | (98.9,99.6) | | |
| Negative Predictive Value (95% CI) | 2.6 | (0.7,6.6) | + | + | + | + | 1.6 | (0.3,4.7) | 2.6 | (0.8,5.9) | | |
| AUC and "accuracy" | 0.6 | low | + | + | + | + | 0.6 | low | 0.6 | low | | |
| Inflation Factor and "Bias" | 0.9 | low | + | + | + | + | 1.0 | low | 0.9 | low | | |

| | | | | | | | | | | | | |
|---|---|---|---|---|---|---|---|---|---|---|---|---|
| **Vaginal births** | | | | | | | | | | | | |
| Observer-assessed coverage % | 98.2 | (96.9,98.9) | 99.8 | (99.3,99.9) | 99.9 | (99.8,100) | 99.8 | (99.7,99.9) | 99.3 | (98.7,99.6) | 99.6 | (99.1,99.9) |
| Survey reported prevalence % | 80.8 | (77.8,83.5) | 85.6 | (83.6,87.3) | 91.1 | (90.4,91.8) | 94.1 | (93.5,94.7) | 92.3 | (90.6,93.8) | 89.3 | (85.3,92.8) |
| "Don't know" responses % | 6.8 | | 1.0 | | 4.3 | | 2.9 | | 2.7 | | 3.2 | (2.0,4.8) |
| **Current survey - Count "don't know" as "no"** | | | | | | | | | | | | |
| > 10 counts in either column of 2x2 table | Yes | | No | | No | | No | | No | | | |
| Percent agreement (TN+TP/n) all observed % | 79.9 | | 85.4 | | 91.1 | | 94.0 | | 92.1 | | 89.1 | (85.0,92.6) |
| Sensitivity % (95% CI) | 80.9 | (77.9,83.7) | + | + | + | + | + | + | + | + | 89.4 | (85.4,92.8) |
| Specificity % (95% CI) | 25.0 | (5.5,57.2) | + | + | + | + | + | + | + | + | 20.4 | (6.4,37.9) |
| Positive Predictive Value % (95% CI) | 98.5 | (97.2,99.3) | + | + | + | + | + | + | + | + | | |
| Negative Predictive Value % (95% CI) | 2.1 | (0.4,6) | + | + | + | + | + | + | + | + | | |
| AUC and "accuracy" | 0.5 | low | + | + | + | + | + | + | + | + | | |
| Inflation Factor and "Bias" | 0.8 | low | + | + | + | + | + | + | + | + | | |

**Consider only "yes" and "no" (exclude don't know)**

| | | | | | | | | | | | | |
|---|---|---|---|---|---|---|---|---|---|---|---|---|
| > 10 counts in either column of 2x2 table | Yes | | No | | No | | No | | No | | | |
| Percent agreement (TN+TP/n) 2x2 table only % | 85.7 | | 86.3 | | 95.1 | | 96.8 | | 94.7 | | 92.4 | (88.3,95.7) |
| Sensitivity (95% CI) | 86.8 | (84,89.2) | + | + | + | + | + | + | + | + | 92.7 | (88.7,95.9) |
| Specificity (95% CI) | 18.2 | (2.3,51.8) | + | + | + | + | + | + | + | + | 15.7 | (3.1,33.1) |
| Positive Predictive Value (95% CI) | 98.5 | (97.2,99.3) | + | + | + | + | + | + | + | + | | |
| Negative Predictive Value (95% CI) | 2.2 | (0.3,7.6) | + | + | + | + | + | + | + | + | | |
| AUC and "accuracy" | 0.5 | low | + | + | + | + | + | + | + | + | | |
| Inflation Factor and "Bias" | 0.9 | low | + | + | + | + | + | + | + | + | | |

| | | | | | | | | | | | | |
|---|---|---|---|---|---|---|---|---|---|---|---|---|
| **Caesarean Births** | | | | | | | | | | | | |
| Observer-assessed coverage % | 99.4 | (98.9,99.6) | 100.0 | - | 99.8 | (99.2,100) | 98.3 | (96.6,99.1) | 99.1 | (98.6,99.5) | 99.5 | (98.9,99.9) |
| Survey reported prevalence % | 80.1 | (78.3,81.7) | 92.0 | (90.1,93.5) | 29.6 | (26.8,32.5) | 50.2 | (44.7,55.6) | 70.8 | (68.5,73) | 66.3 | (44.0,85.3) |
| "Don't know" responses % | 17.1 | | 3.7 | | 59.3 | | 42.4 | | 20.5 | | 26.0 | (10.1,46.1) |
| **Current survey - Count "don't know" as "no"** | | | | | | | | | | | | |
| > 10 counts in either column of 2x2 table | Yes | | No | | No | | No | | No | | | |
| Percent agreement (TN+TP/n) all observed % | 79.7 | | 92.0 | | 31.0 | | 52.0 | | 70.5 | | 66.7 | (45.3,85.0) |
| Sensitivity % (95% CI) | 80.1 | (78.3,81.8) | + | + | + | + | + | + | + | + | 66.6 | (45.1,85.0) |
| Specificity % (95% CI) | 30.8 | (9.1,61.4) | + | + | + | + | + | + | + | + | 62.8 | (16.1,99.1) |

| | | | | | | | | | | | | |
|---|---|---|---|---|---|---|---|---|---|---|---|---|
| Positive Predictive Value % (95% CI) | 99.5 | (99,99.8) | + | + | + | + | + | + | + | + | | |
| Negative Predictive Value % (95% CI) | 1.0 | (0.3,2.4) | + | + | + | + | + | + | + | + | | |
| AUC and "accuracy" | 0.6 | low | + | + | + | + | + | + | + | + | | |
| Inflation Factor and "Bias" | 0.8 | low | + | + | + | + | + | + | + | + | | |

**Consider only "yes" and "no" (exclude don't know)**

| | | | | | | | | | | | | |
|---|---|---|---|---|---|---|---|---|---|---|---|---|
| > 10 counts in either column of 2x2 table | Yes | | No | | No | | No | | No | | | |
| Percent agreement (TN+TP/n) 2x2 table only % | 96.1 | | 95.5 | | 74.2 | | 88.1 | | 88.6 | | 89.7 | (82.5,95.2) |
| Sensitivity (95% CI) | 96.6 | (95.7,97.4) | + | + | + | + | + | + | + | + | 90.0 | (82.5,95.5) |
| Specificity (95% CI) | 18.2 | (2.3,51.8) | + | + | + | + | + | + | + | + | 32.5 | (0.2,78.4) |
| Positive Predictive Value (95% CI) | 99.5 | (99,99.8) | + | + | + | + | + | + | + | + | | |
| Negative Predictive Value (95% CI) | 3.3 | (0.4,11.5) | + | + | + | + | + | + | + | + | | |
| AUC and "accuracy" | 0.6 | low | + | + | + | + | + | + | + | + | | |
| Inflation Factor and "Bias" | 1.0 | low | + | + | + | + | + | + | + | + | | |

| **Don't Know Responses** |
|---|
| >20% : Poor |
| 15.1-20 % : Moderate |
| 10.1-15 % : Good |
| 5.1-10 % :  Very Good |
| 0-5 % : Excellent |

N= 23,015 women observer-assessed to give birth
CI=confidence interval
HMIS= health management information system
AUC= Area under the curve
+=result suppressed due to 10 or fewer count per column of two-by-two table for some results.
As reported in an associated paper [1]

**Reference**

1. Day L, Rahman QS, Rahman A, Salim N, KC A, Ruysen H, Tahsina T, Masanja H, Basnet O, Gore-langton G *et al*: **Assessment of the validity of the measurement of newborn and maternal health-care coverage in hospitals (EN-BIRTH): a mixed-methods observational study** *Lancet Global* (2020) DOI: 10.1016/S2214-109X(20)30504-0.
