# Supplementary material for: Uterotonics for prevention of postpartum haemorrhage: EN-BIRTH multi-country validation study
Source: BMC Pregnancy Childbirth. 2021 Mar 26;21(Suppl 1):230. doi: 10.1186/s12884-020-03420-x (PMC7995712; doi:10.1186/s12884-020-03420-x)
Supplement: Supplementary file 10 — Additional file 10. Individual-level validation of register recording for uterotonic administration, EN-BIRTH study (n = 15,645). [file 12884_2020_3420_MOESM10_ESM.pdf]

Every Newborn BIRTH multi-country validation study: informing measurement of coverage and quality of maternal and newborn care

Uterotonics for prevention of postpartum haemorrhage: EN-BIRTH multi-country validation study

Additional File 10: Individual-level validation of register recording for uterotonic administration, EN-BIRTH study (n=15,645)

	Bangladesh				Nepal		Tanzania				All sites Pooled	
	Azimpur Tertiary		Kustia District		Pokhara Regional		Temeke Regional		Azimpur National		Random Effects	
	%	95% CI	%	95% CI	%	95% CI	%	95% CI	%	95% CI	%	95% CI
**All Modes Of Birth**												
Observer-assessed Coverage %	98.9	(98.4,99.2)	99.8	(99.5,99.9)	N/A	N/A	99.3	(99.1,99.5)	98.4	(98.0,98.8)	99.2	(98.6,99.7)
Register recorded prevalence %	99.4	(99,99.7)	21.6	(19.9,23.4)	N/A	N/A	97.6	(97.2,97.9)	64.5	(62.9,66)	77.9	(37.8,99.5)
Not recorded %	0.0		0.0		N/A		0.8		31.4		3.1	(0.0,19.1)
Not readable %	0.0		0.1		N/A		0.1		0.1		0.1	(0.0,0.1)
**Current HMIS - count "not recorded" as "no"**												
> 10 counts in either column of 2x2 table	Yes		No		N/A		Yes		Yes			
Percent agreement (TN+TP/n) all observed %	99.0		21.6		N/A		97.3		64.5		77.2	(37.7,99.3)
Sensitivity % (95% CI)	99.5	(99,99.7)	+	+	N/A	N/A	97.6	(97.2,98)	64.6	(63,66.2)	78.0	(37.8,99.5)
Specificity % (95% CI)	10.0	(0.3,44.5)	+	+	N/A	N/A	8.3	(1,27)	51.7	(32.5,70.6)	22.8	(1.7,53.6)
Positive Predictive Value % (95% CI)	99.6	(99.2,99.8)	+	+	N/A	N/A	99.7	(99.5,99.8)	99.4	(99,99.7)		
Negative Predictive Value % (95% CI)	7.7	(0.2,36)	+	+	N/A	N/A	1.3	(0.2,4.6)	1.2	(0.7,2)		
AUC and "accuracy"	0.6	low	+	+	N/A	N/A	0.5	low	0.6	low		
Inflation Factor and "Bias"	1.0	low	+	+	N/A	N/A	1.0	low	0.6	Moderate		
**Consider only "given" and "not given" (exclude not recorded)**												
> 10 counts in either column of 2x2 table	Yes		No		N/A		Yes		Yes			
Percent agreement (TN+TP/n) 2x2 table only %	99.1		21.6		N/A		98.1		93.6		86.1	(48.5,100.0)
Sensitivity % (95% CI)	99.5	(99.1,99.7)	+	+	N/A	N/A	98.4	(98.1,98.7)	94.1	(93.1,95)	3.5	(0.0,17.2)
Specificity % (95% CI)	10.0	(0.3,44.5)	+	+	N/A	N/A	0.0	(0,15.4)	6.7	(0.2,31.9)	85.2	(48.1,100.0)
Positive Predictive Value % (95% CI)	99.6	(99.2,99.8)	+	+	N/A	N/A	99.7	(99.5,99.8)	99.4	(99,99.7)		
Negative Predictive Value % (95% CI)	8.3	(0.2,38.5)	+	+	N/A	N/A	0.0	(0,3.6)	0.7	(0,3.9)		
AUC and "accuracy"	0.6	low	+	+	N/A	N/A	0.5	low	0.5	low		
Inflation Factor and "Bias"	1.0	low	+	+	N/A	N/A	1.0	low	0.9	low		

**Vaginal Births**												
Observer-assessed coverage %	98.2	(96.9,98.9)	99.8	(99.3,99.9)	99.9	(99.8,100)	99.8	(99.7,99.9)	99.3	(98.7,99.6)	99.4	(98.7,99.9)
Register recorded prevalence %	98.9	(97.6,99.5)	32.8	(30.2,35.5)	N/A	N/A	97.6	(97.2,97.9)	92.5	(91,93.7)	86.6	(55.0,100.0)
Not recorded %	0.0		0.0		N/A		0.8		500.0		0.7	(0.0,2.9)
Not readable %	0.2		0.1		N/A		0.1		0.0		0.0	(0.0,0.1)
**Current HMIS - count "not recorded" as "no"**												
> 10 counts in either column of 2x2 table	No		No		N/A		Yes		No			
Percent agreement (TN+TP/n) all observed %	98.4		32.7		N/A		97.4		91.9		85.9	(54.4,100.0)
Sensitivity % (95% CI)	98.9	(97.7,99.6)	+	+	N/A	N/A	97.6	(97.2,98)	+	+	86.5	(54.9,100.0)
Specificity % (95% CI)	0.0	(0,70.8)	+	+	N/A	N/A	9.1	(0.2,41.3)	+	+	3.6	(0.0,19.1)
Positive Predictive Value % (95% CI)	99.5	(98.4,99.9)	+	+	N/A	N/A	99.8	(99.7,99.9)	+	+		
Negative Predictive Value % (95% CI)	0.0	(0,45.9)	+	+	N/A	N/A	0.7	(0,3.8)	+	+		
AUC and "accuracy"	0.5	low	+	+	N/A	N/A	0.5	low	+	+		
Inflation Factor and "Bias"	1.0	low	+	+	N/A	N/A	1.0	low	+	+		
**Consider only "given" and "not given" (exclude not recorded)**												
> 10 counts in either column of 2x2 table	No		No		N/A		Yes		No			
Percent agreement (TN+TP/n) 2x2 table only %	98.6		32.7		N/A		98.3		96.8		88.3	(56.2,100.0)
Sensitivity % (95% CI)	+	+	+	+	N/A	N/A	98.4	(98.1,98.7)	+	+	89.0	(56.9,100.0)
Specificity % (95% CI)	+	+	+	+	N/A	N/A	0.0	(0,30.8)	+	+	0.2	(0.0,11.5)
Positive Predictive Value % (95% CI)	+	+	+	+	N/A	N/A	99.8	(99.7,99.9)	+	+		
Negative Predictive Value % (95% CI)	+	+	+	+	N/A	N/A	0.0	(0,3.8)	+	+		
AUC and "accuracy"	+	+	+	+	N/A	N/A	0.5	low	+	+		
Inflation Factor and "Bias"	+	+	+	+	N/A	N/A	1.0	low	+	+		
**Caesarean Births**												
Observer-assessed coverage %	99.4	(98.9,99.6)	100.0	(100,100)	99.8	(99.2,100)	98.3	(96.6,99.1)	99.1	(98.6,99.5)	99.4	(98.7,99.9)
Register recorded prevalence %	99.6	(99.2,99.8)	6.0	(4.5,7.7)	N/A	N/A	98.0	(96.2,99)	43.1	(41,45.3)	68.5	(15.5,100.0)
Not recorded %	0.0		0.0		N/A		0.4		51.5		5.0	(0.0,39.1)
Not readable %	0.0		0.1		N/A		0.0		0.2		0.1	(0.0,0.2)
**Current HMIS - count "not recorded" as "no"**												
> 10 counts in either column of 2x2 table	No		No		N/A		No		Yes			
Percent agreement (TN+TP/n) all observed %	99.3		6.0		N/A		96.9		43.5		67.2	(15.6,99.7)
Sensitivity % (95% CI)	+	+	+	+	N/A	N/A	+	+	43.3	(41.1,45.5)	68.5	(15.5,100.0)

Specificity % (95% CI)	+	+	+	+	N/A	N/A	+	+	75.0	(42.8,94.5)	18.5	(0.0,82.1)
Positive Predictive Value % (95% CI)	+	+	+	+	N/A	N/A	+	+	99.4	(98.9,99.7)		
Negative Predictive Value % (95% CI)	+	+	+	+	N/A	N/A	+	+	0.8	(0.4,1.5)		
AUC and "accuracy"	+	+	+	+	N/A	N/A	+	+	0.6	low		
Inflation Factor and "Bias"	+	+	+	+	N/A	N/A	+	+	0.4	Large		

Consider only "given" and "not given" (exclude not recorded)

> 10 counts in either column of 2x2 table	No		No		N/A		No		No			
Percent agreement (TN+TP/n) 2x2 table only %	99.3		6.0		N/A		97.4		89.1		78.8	(25.1,100.0)
Sensitivity % (95% CI)	+	+	+	+	N/A	N/A	+	+	+	+	80.1	(25.5,100.0)
Specificity % (95% CI)	+	+	+	+	N/A	N/A	+	+	+	+	2.7	(0.0,22.6)
Positive Predictive Value % (95% CI)	+	+	+	+	N/A	N/A	+	+	+	+		
Negative Predictive Value % (95% CI)	+	+	+	+	N/A	N/A	+	+	+	+		
AUC and "accuracy"	+	+	+	+	N/A	N/A	+	+	+	+		
Inflation Factor and "Bias"	+	+	+	+	N/A	N/A	+	+	+	+		

N= 15,645 (all women observer-assed to give birth in Tanzania, and those during use of the revised register in Bangladesh).
Pokhara excluded as has no column in the register.
CI=confidence interval
HMIS= health management information system
AUC= Area under the curve
N/A=data element not captured by routine register
+=result suppressed due to 10 or fewer count per column of two-by-two table
As reported in an associated paper [1]

Reference

1. Day L, Rahman QS, Rahman A, Salim N, KC A, Ruysen H, Tahsina T, Masanja H, Basnet O, Gore-langton G *et al*: **Assessment of the validity of the measurement of newborn and maternal health-care coverage in hospitals (EN-BIRTH): a mixed-methods observational study** *Lancet Global* (2020) DOI: 10.1016/S2214-109X(20)30504-0.
