# Supplementary material for: Uterotonics for prevention of postpartum haemorrhage: EN-BIRTH multi-country validation study
Source: BMC Pregnancy Childbirth. 2021 Mar 26;21(Suppl 1):230. doi: 10.1186/s12884-020-03420-x (PMC7995712; doi:10.1186/s12884-020-03420-x)
Supplement: Supplementary file 11 — Additional file 11. Comparison of uterotonic coverage measurement using original and revised Bangladesh registers, EN-BIRTH study (n = 5207). [file 12884_2020_3420_MOESM11_ESM.pdf]

Every Newborn BIRTH multi-country validation study: informing measurement of coverage and quality of maternal and newborn care

Uterotonics for prevention of postpartum haemorrhage: EN-BIRTH multi-country validation study

Additional File 11: Comparison of uterotonic coverage measurement using original and revised Bangladesh registers, EN-BIRTH study (n=5,207)

	**Old Register**				**New Register**			
	%	SD	95% CI		%	SD	95% CI	
**Data Extraction**								
Azimpur	17.8	1.5	15.1	20.9	99.4	0.2	99.0	99.7
Kushtia	0.0	-	-	-	21.6	0.9	19.9	23.4
**Data Extraction : NVD/Forceps/ Vacuum**								
Azimpur	0.0	-	-	-	98.9	0.4	97.6	99.5
Kushtia	97.6	1.2	93.7	99.1	32.8	1.4	30.2	35.5
**Data Extraction : Caesarean section (Elective/ Emergency)**								
Azimpur	25.5	2.0	21.7	29.6	99.6	0.1	99.2	99.8
Kushtia	0.0	-	-	-	6.0	0.8	4.5	7.7

N= 5,207 register recorded cases from original and revised registers in Bangladesh.
NVD= normal vaginal delivery
SD= Standard deviation
CI= Confidence interval
Register recorded uterotonic administration increased in both hospitals with roll out of new registers including a specific column for uterotonic documentation, although not equally.
Register recorded cases increased more in Azimpur than Kushtia.
