# Supplementary material for: Uterotonics for prevention of postpartum haemorrhage: EN-BIRTH multi-country validation study
Source: BMC Pregnancy Childbirth. 2021 Mar 26;21(Suppl 1):230. doi: 10.1186/s12884-020-03420-x (PMC7995712; doi:10.1186/s12884-020-03420-x)
Supplement: Supplementary file 12 — Additional file 12. Association testing for timing of Oxytocin administration, EN-BIRTH Study (n = 22,121). [file 12884_2020_3420_MOESM12_ESM.pdf]

Every Newborn BIRTH multi-country validation study: informing measurement of coverage and quality of maternal and newborn care

Uterotonics for prevention of postpartum haemorrhage: EN-BIRTH multi-country validation study

Additional File 12: Association testing for timing of Oxytocin administration, EN-BIRTH Study (n=22,121)

Uterotonics Timing[1]	Odds Ratio	Std. Err.	P-value	95% CI
**By Mode of Birth[2]**				
<1 min	0.061	0.008	0.000	0.048 - 0.078
1<3 mins	0.209	0.025	0.000	0.165- 0.264
3-<6 mins	0.743	0.095	0.020	0.578 - 0.954
6-<9mins	1.139	0.188	0.429	0.825 - 1.574
**By Route of Administration (IM)**				
<1 min	0.052	0.006	0.000	0.042 -0.065
1<3 mins	0.213	0.023	0.000	0.172-0.263
3-<6 mins	0.711	0.081	0.003	0.568-0.889
6-<9mins	1.082	0.157	0.586	0.814-1.439

N=22,121 (women observer-assessed to receive oxytocin from 0 to 30 minutes after birth)
IM= intramuscular
[1]Reference >9 minutes
[2]Caesarean and vaginal births used as reference group
Assessed using univariate logistical regression test of association.
