# Supplementary material for: Uterotonics for prevention of postpartum haemorrhage: EN-BIRTH multi-country validation study
Source: BMC Pregnancy Childbirth. 2021 Mar 26;21(Suppl 1):230. doi: 10.1186/s12884-020-03420-x (PMC7995712; doi:10.1186/s12884-020-03420-x)
Supplement: Supplementary file 13 — Additional file 13. Oxytocin dose by EN-BIRTH site and mode of birth, EN-BIRTH study (n = 22,269). [file 12884_2020_3420_MOESM13_ESM.pdf]

Every Newborn BIRTH multi-country validation study: informing measurement of coverage and quality of maternal and newborn care

Uterotonics for prevention of postpartum haemorrhage: EN-BIRTH multi-country validation study

Additional File 13: Oxytocin dose by EN-BIRTH site and mode of birth, EN-BIRTH study

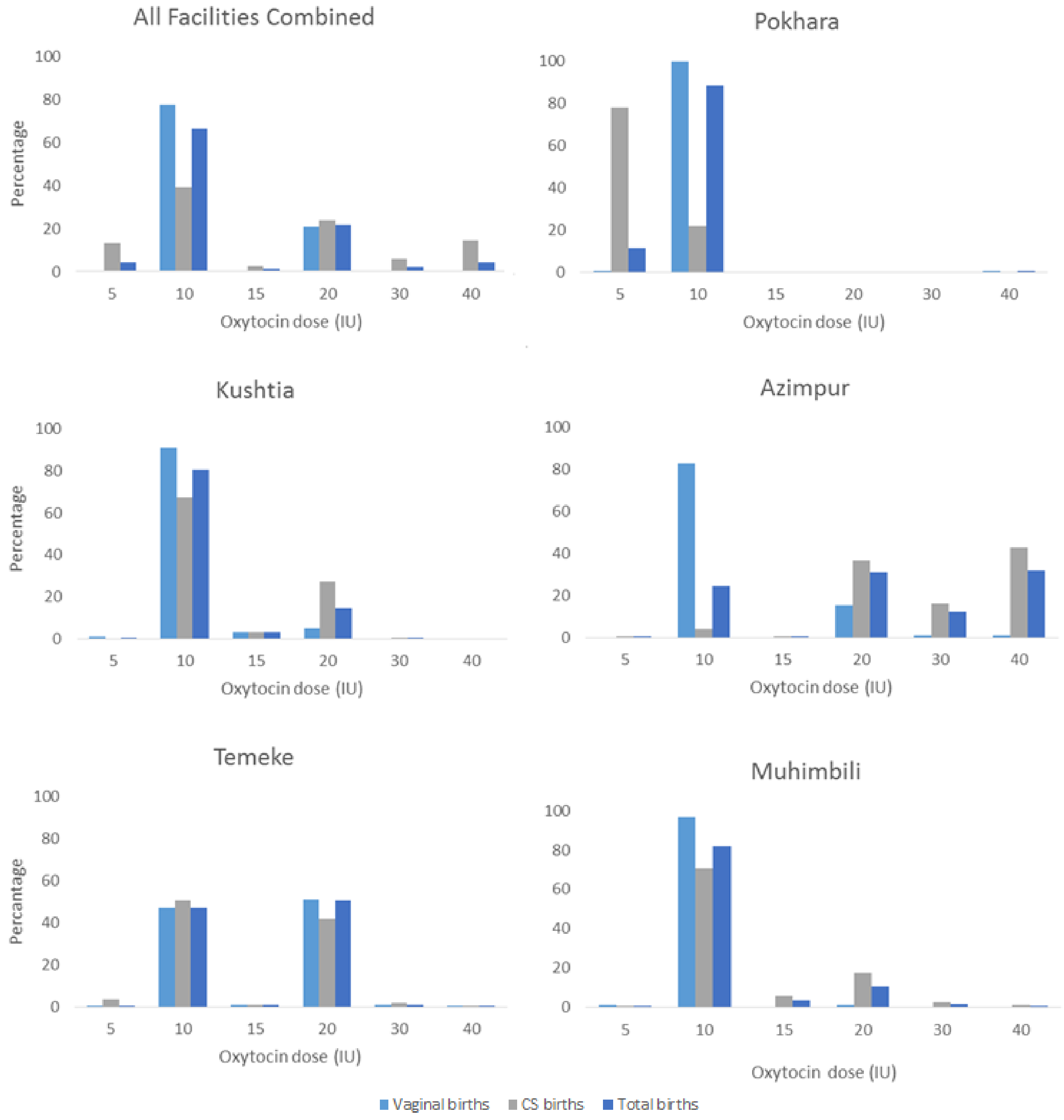

(n=22,269)

N= 22,269 women observed to receive oxytocin.
IU: international units
This is descriptive data therefore total column is based on individually weighted averages.
