# Supplementary material for: Uterotonics for prevention of postpartum haemorrhage: EN-BIRTH multi-country validation study
Source: BMC Pregnancy Childbirth. 2021 Mar 26;21(Suppl 1):230. doi: 10.1186/s12884-020-03420-x (PMC7995712; doi:10.1186/s12884-020-03420-x)
Supplement: Supplementary file 14 — Additional file 14. Estimated Blood Loss (EBL) compared with Oxytocin coverage, EN-BIRTH Study. [file 12884_2020_3420_MOESM14_ESM.pdf]

Every Newborn BIRTH multi-country validation study: informing measurement of coverage and quality of maternal and newborn care

Uterotonics for prevention of postpartum haemorrhage: EN-BIRTH multi-country validation study

Additional File 14: Estimated Blood Loss (EBL) compared with Oxytocin coverage, EN-BIRTH Study

	Bangladesh		Nepal	Tanzania		Total*
	Azimpur Tertiary	Kushtia District	Pokhara Regional	Temeke Regional	Muhimbili National	
	n(%)	n(%)	n(%)	n(%)	n(%)	n(%)
**EBL for women who received 40IU Oxytocin**						
**Total**	**900(100)**	**0(0.0)**	**2(100)**	**5(100)**	**31(100)**	**938(100)**
**All Modes of Birth Combined**						
Normal: ≤500 mls	876(97.3)	0(0.0)	2(100.0)	5(100.0)	22(70.9)	905(96.5)
PPH: >500 - ≤1000 mls	13(1.4)	0(0.0)	0(0.0)	5(100.0)	8(25.8)	21(2.2)
Severe PPH > 1000 mls	3(0.3)	0(0.0)	0(0.0)	5(100.0)	0(0.0)	3(0.3)
Missing	8(0.9)	0(0.0)	0(0.0)	5(100.0)	1(3.2)	9(0.9)
**Vaginal Births**						
Normal: ≤500 mls	7(100.0)	0(0.0)	2(100.0)	4(100.0)	2(100.0)	15(100.0)
PPH: >500 - ≤1000 mls	0(0.0)	0(0.0)	0(0.0)	0(0.0)	0(0.0)	0(0.0)
Severe PPH > 1000 mls	0(0.0)	0(0.0)	0(0.0)	0(0.0)	0(0.0)	0(0.0)
Missing	0(0.0)	0(0.0)	0(0.0)	0(0.0)	0(0.0)	0(0.0)
**Caesarean Births**						
Normal: ≤500 mls	869(97.3)	0(0.0)	0(0.0)	1(100.0)	20(71.4)	890(96.5)
PPH: >500 - ≤1000 mls	13(1.5)	0(0.0)	0(0.0)	0(0.0)	7(25.0)	20(2.1)
Severe PPH > 1000 mls	3(0.3)	0(0.0)	0(0.0)	0(0.0)	0(0.0)	3(0.3)
Missing	8(0.9)	0(0.0)	0(0.0)	0(0.0)	1(3.6)	9(0.9)
**EBL for women who received Oxytocin within 0-3 mins of birth**						
**Total**	**2652(100)**	**2164(100)**	**5691(100)**	**4563(100)**	**2944(100)**	**18014(100)**
**All Modes of Birth Combined**						
Normal: ≤500 mls	2578(97.2)	2078(96.0)	5524(97.1)	4393(96.3)	2670(90.7)	17243(95.7)
PPH: >500 - ≤1000 mls	46(1.7)	57(2.6)	92(1.6)	102(2.2)	204(6.9)	501(2.8)
Severe PPH > 1000 mls	6(0.2)	8(0.4)	1(0.02)	4(0.1)	10(0.3)	29(0.2)
Missing	22(0.8)	21(0.9)	74(1.3)	64(1.4)	60(2.0)	241(1.3)
**Vaginal Births**						
Normal: ≤500 mls	624(96.3)	1197(96.0)	4568(96.8)	3980(97.2)	1037(95.1)	11406(96.8)
PPH: >500 - ≤1000 mls	20(3.1)	41(3.3)	79(1.7)	45(1.1)	36(3.3)	221(1.9)
Severe PPH > 1000 mls	2(0.3)	5(0.4)	1(0.02)	4(0.1)	2(0.2)	14(0.1)
Missing	2(0.3)	4(0.3)	71(1.5)	44(1.1)	15(1.4)	136(1.2)
**Caesarean Births**						
Normal: ≤500 mls	1951(97.6)	877(96.1)	951(98.3)	334(83.9)	1533(88.2)	5646(93.9)
PPH: >500 - ≤1000 mls	25(1.3)	16(1.7)	13(1.3)	56(14.1)	162(9.3)	272(4.5)
Severe PPH > 1000 mls	4(0.2)	3(0.3)	0(0.0)	0(0.0)	8(0.5)	15(0.2)
Missing	19(0.9)	17(1.9)	3(0.3)	8(2.0)	35(2.0)	82(1.4)

Estimated blood loss (EBL) was assessed via visual observation which can be inaccurate, especially for caesarean sections where blood loss is often underestimated.
*Descriptive data: total column is therefore based on individually weighted averages.
