# Supplementary material for: Uterotonics for prevention of postpartum haemorrhage: EN-BIRTH multi-country validation study
Source: BMC Pregnancy Childbirth. 2021 Mar 26;21(Suppl 1):230. doi: 10.1186/s12884-020-03420-x (PMC7995712; doi:10.1186/s12884-020-03420-x)

Every Newborn BIRTH multi-country validation study: informing measurement of coverage and quality of maternal and newborn care

Uterotonics for prevention of postpartum haemorrhage: EN-BIRTH multi-country validation study

Additional File 15: Assessment of routine recording responsibilities for uterotonic provision, EN-BIRTH Study

a. As reported by EN-BIRTH data collectors

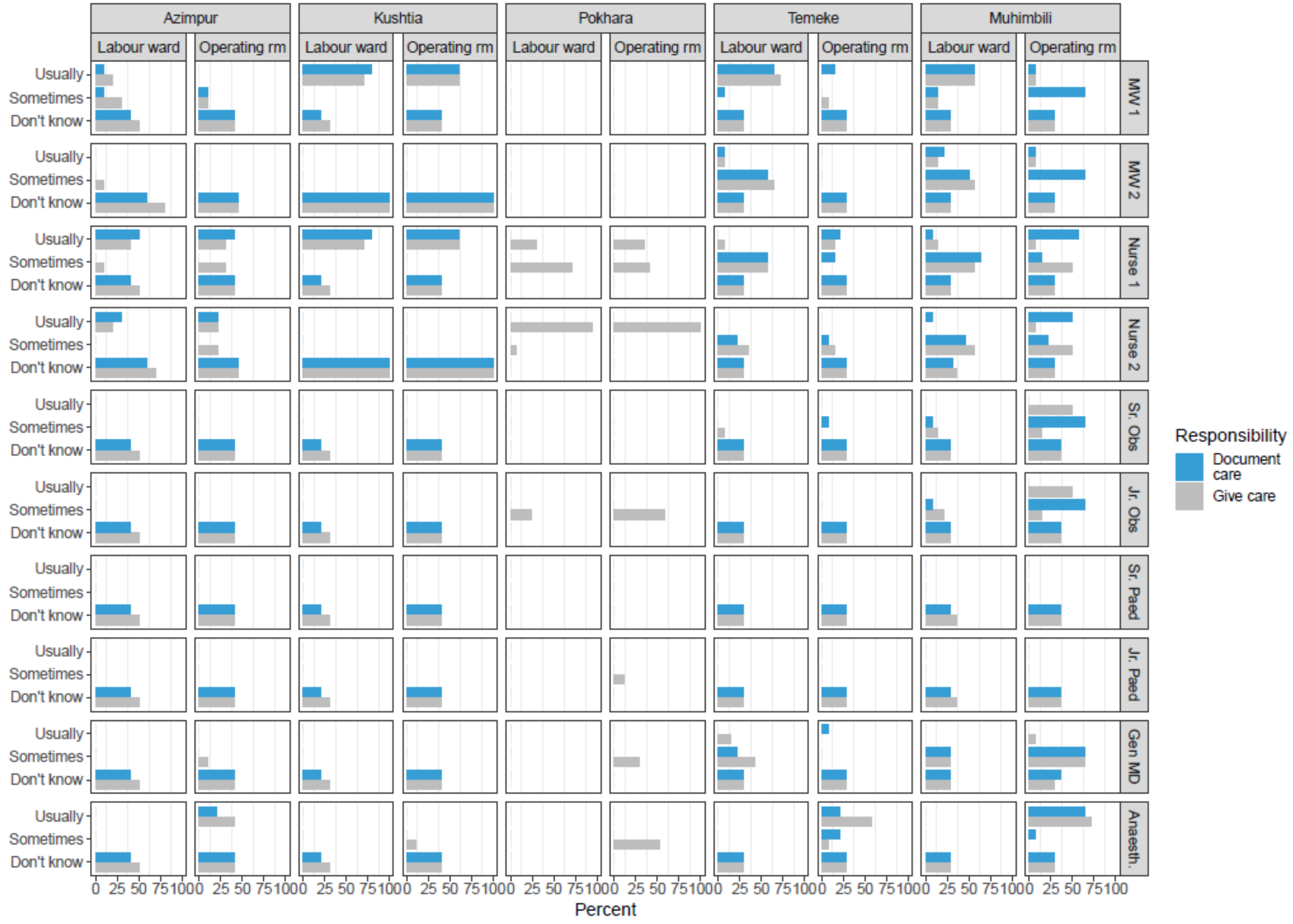

b. As reported by health workers

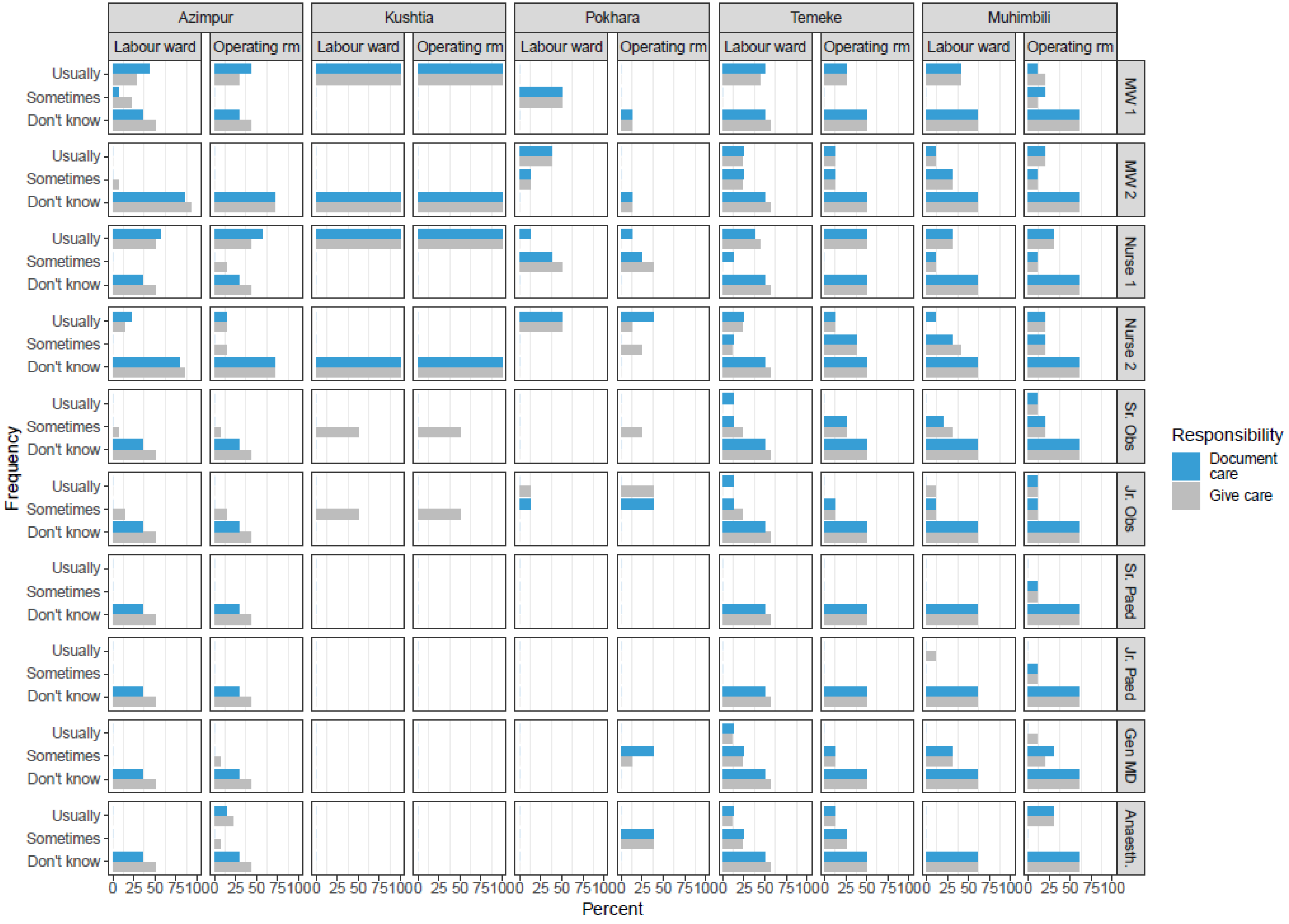

Supplement: Supplementary file 15 — Additional file 15. Assessment of routine recording responsibilities for uterotonic provision, EN-BIRTH Study. [file 12884_2020_3420_MOESM15_ESM.pdf]
