# Supplementary material for: Uterotonics for prevention of postpartum haemorrhage: EN-BIRTH multi-country validation study
Source: BMC Pregnancy Childbirth. 2021 Mar 26;21(Suppl 1):230. doi: 10.1186/s12884-020-03420-x (PMC7995712; doi:10.1186/s12884-020-03420-x)

Every Newborn BIRTH multi-country validation study: informing measurement of coverage and quality of maternal and newborn care

Uterotonics for prevention of postpartum haemorrhage: EN-BIRTH multi-country validation study

Additional File 16: Register recording order and prioritisation for uterotonic provision, EN-BIRTH study

a. As reported by EN-BIRTH data collectors

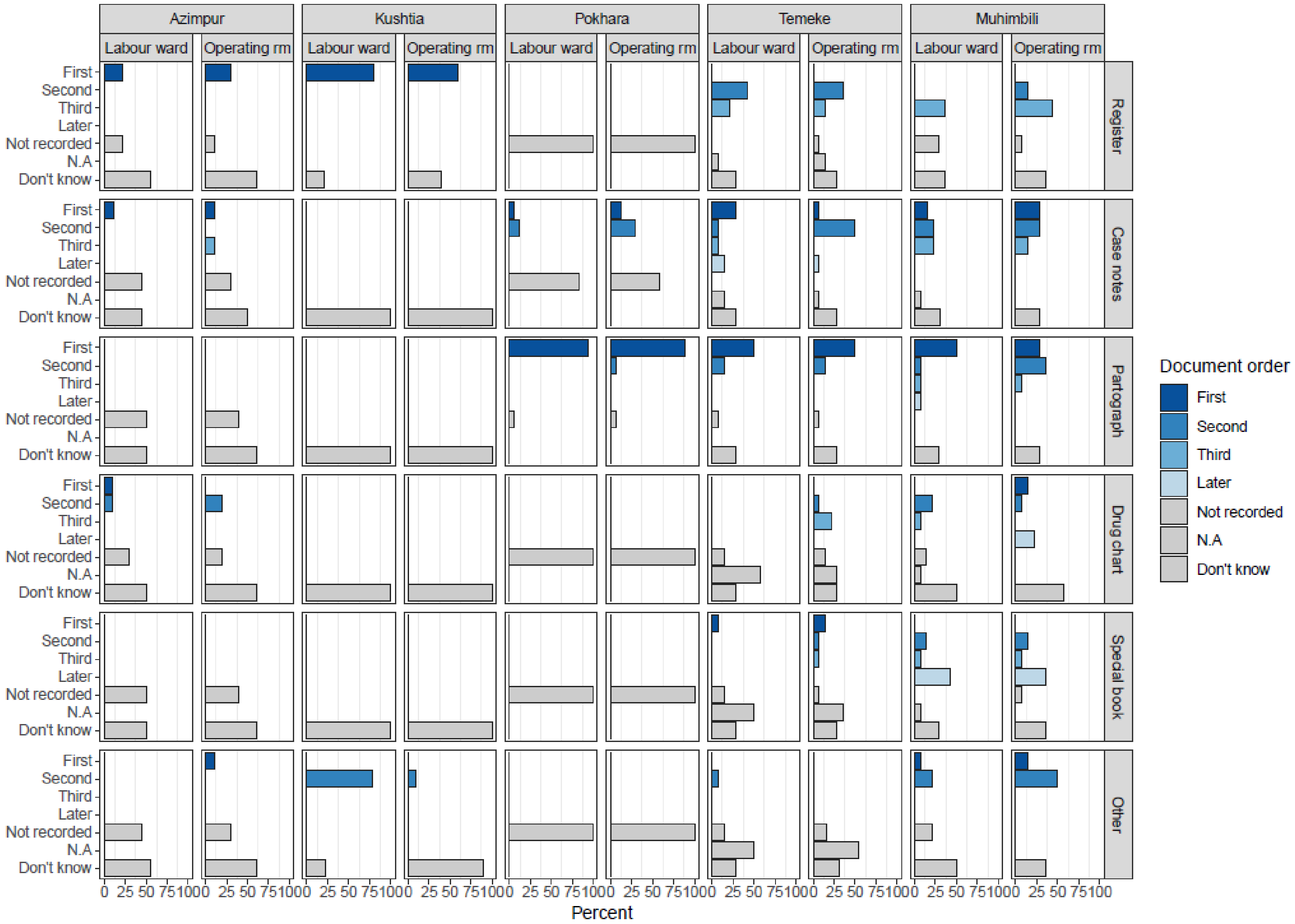

b. As reported by health workers

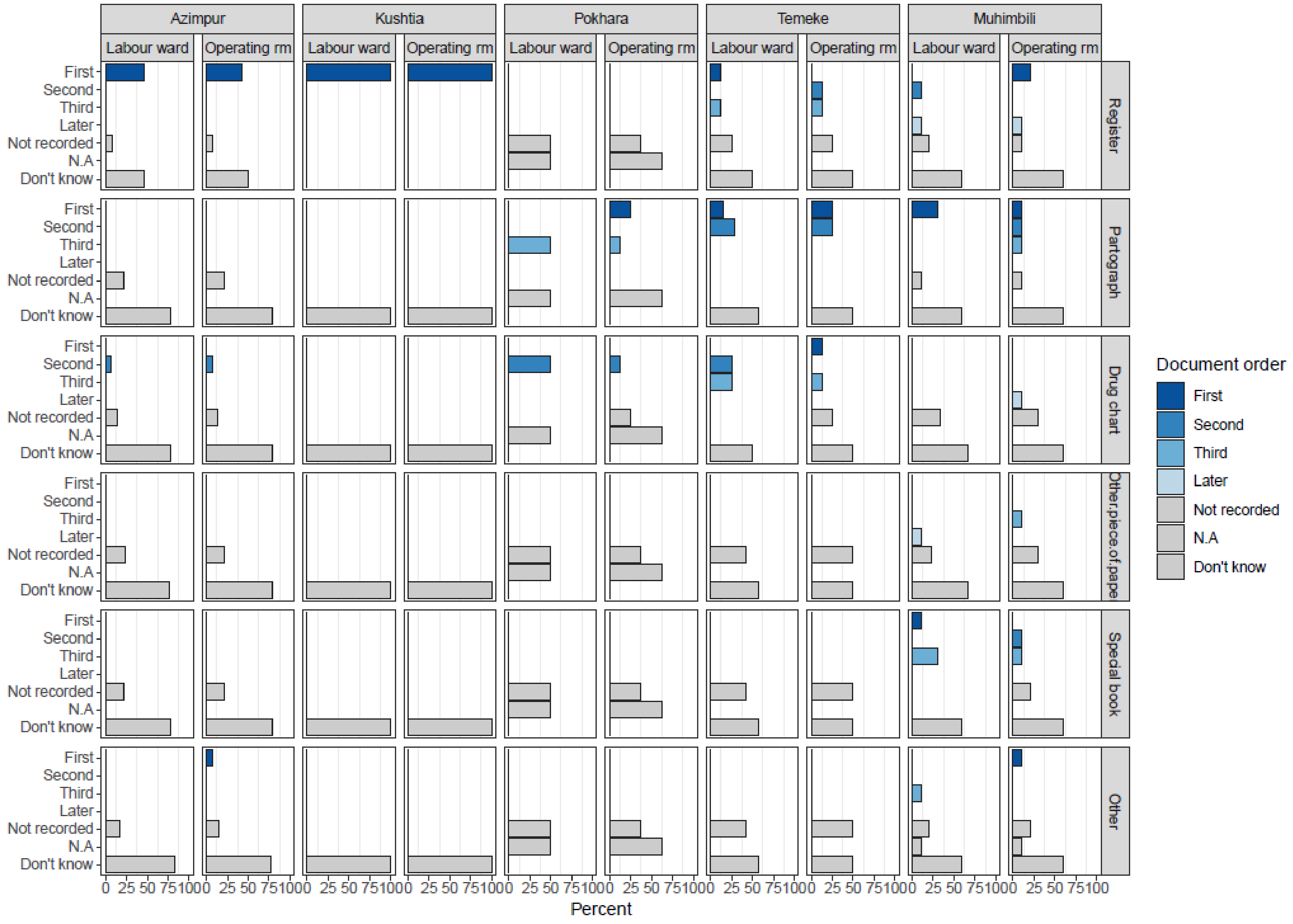

Supplement: Supplementary file 16 — Additional file 16. Register recording order and prioritisation for uterotonic provision, EN-BIRTH study. [file 12884_2020_3420_MOESM16_ESM.pdf]
